# Heteronuclear Complexes Containing Pt(II) and Ag(I) Centers: Application to Efficient Light-Emitting Electrochemical Cells

**DOI:** 10.1021/acsami.4c22938

**Published:** 2025-05-19

**Authors:** Ariadna Lázaro, Margarita Crespo, Piotr Pander, Fernando B. Dias, Laura Rodríguez

**Affiliations:** † Departament de Química Inorgànica i Orgànica, Secció de Química Inorgànica, 16724 Universitat de Barcelona, Martí i Franquès 1-11, E-08028 Barcelona, Spain; ‡ Institut de Nanociència i Nanotecnologia (IN2UB), 16724 Universitat de Barcelona, 08028 Barcelona, Spain; § Institut de Biomedicina de la Universitat de Barcelona (IBUB), 08028 Barcelona, Spain; ∥ Faculty of Chemistry, 366466 Silesian University of Technology, M. Strzody 9, 44-100 Gliwice, Poland; ⊥ Centre for Organic and Nanohybrid Electronics, 366466 Silesian University of Technology, Konarskiego 22B, 44-100 Gliwice, Poland; # Department of Physics, 3057 Durham University, South Road, Durham DH1 3LE, U.K.

**Keywords:** platinum complex, heteronuclear complex, cyclometalation, LEECs, photophysics

## Abstract

We report the synthesis of novel ionic heteronuclear Pt­(II)–Ag­(I) complexes derived from Pt­(NCN)-C≡CR precursors (R = aryl), where Ag^+^ ions coordinate to the acetylene groups. The photophysical investigation reveals a complex interplay of emissions: ^3^MLCT and ^3^LC states from the Pt­(NCN) units, ^3^LC emissions from the R aryl groups, and ^3^MMLCT emissions arising from aggregated Pt­(NCN)-X units in the solid state. These complexes exhibit photoluminescence in the range of 650–750 nm, predominantly from ^3^MMLCT states facilitated by short Pt···Pt contacts. Utilizing complex 4c (R = phenanthryl) as the ionic emitter in proof-of-concept LEECs, we achieved a maximum EQE of 4.1% and a luminance of nearly 2000 cd m^–2^. These results represent one of the highest-performing LEECs incorporating Pt­(II)-based phosphorescent complexes, underscoring their potential in light-emitting applications.

## Graphical abstract

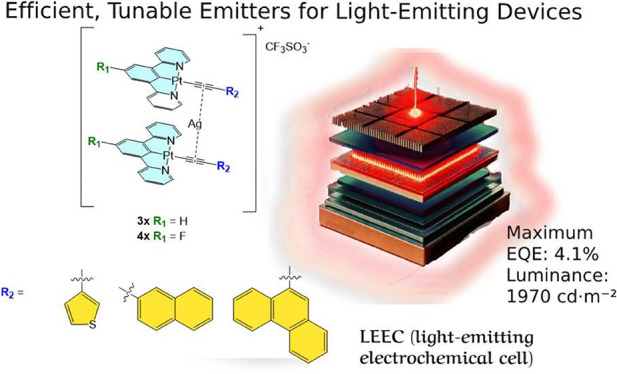

## Introduction

Platinum­(II) complexes are among the most studied transition metal compounds, second only to iridium­(III)-based luminophores in popularity. The d^8^ electronic configuration of the Pt^2+^ ion gives rise to a characteristic square planar geometry in its complexes. A notable feature of this geometry is the propensity to form metallophilic Pt···Pt short contacts, either in the ground or excited state. These interactions often result in the generation of long-wavelength emissive triplet metal–metal-to-ligand charge-transfer (^3^MMLCT) states, making such complexes valuable for near-infrared (NIR) luminescence applications. Applications of NIR light and luminophores include biological imaging,^1^ photodynamic therapy,^2^ wearable sensors for health monitoring,^3^ night-vision^4^ and security authentication devices, such as fingerprint sensors.^5^ Platinum­(II) complexes are also widely utilized as monomeric visible-light emitters in organic light-emitting diodes (OLEDs). Despite being studied for decades, these complexes continue to reveal surprising properties, particularly when their luminescence is investigated in-depth and with meticulous detail.^6^

The production of commercial OLEDs through thermal evaporation generally requires large vacuum chambers and is highly material-intensive, with less than 5% of the material being incorporated into the final device. While this technique is widely employed in various electronic devices, its application is primarily limited to high-end products such as televisions and mobile phones. For low-end applications, where high quality and long-term stability are less critical, solution-based techniques offer a more practical alternative. A related but simpler technology is solution-processed light-emitting electrochemical cells (LEECs or LECs), first introduced in 1995 by Pei and colleagues.^7^ LEECs often use a mixture of conjugated luminescent materials and a conductive polymer in an ionic environment as the emissive layer.^8,9^ Instead of multiple layers, LEECs require only one layer sandwiched between contact electrodes to operate. This simplicity is beneficial as it also allows reducing the environmental and financial burdens of producing (low-end) electronic devices in general.^10,11^

LEECs using ionic transition metal complexes (iTMCs) have become of particular interest in recent years, following the success of charge neutral complexes in OLEDs.^12–17^ Despite the popularity of iTMC-based LEECs, metal-free emitters are also being investigated.^18^ iTMCs used in LEECs are most commonly based on iridium,^11,15,19–27^ although some examples using other transition metals such as gold,^28^ ruthenium,^8,29–31^ rhenium^9^ or copper^10^ have also been developed. Nevertheless, to the best of our knowledge, the use of ionic platinum complexes in LEECs has not yet been widely explored. For these reasons and due to our earlier experiences with luminescent platinum complexes,^32–34^ we have decided to study this topic more in-depth.

In our work, we have combined the strongly luminescent properties of Pt­(NCN)-X complexes (where X = C≡C-R) with Ag^+^ ions coordinating to the acetylene groups. Our findings indicate that Ag^+^ ions bind two Pt­(NCN)-X units together, forming unprecedented ionic trimetallic heteronuclear complexes. These complexes were employed as ionic emitters in proof-of-concept LEECs, achieving external quantum efficiencies (EQE) of up to 4.1% and a maximum luminance of nearly 2000 cd m^–2^.

## Experimental Section

### General Procedures

All reagents were obtained from commercial sources and used as received. Complexes Pt­(dpyb)Cl (**1**), Pt­(dpyb-F)­Cl, (**2**) Pt­(dpyb)-C≡CThio (**1a**), Pt­(dpyb-F)-C≡CThio (**2a**), Pt­(dpyb)-C≡CNaph (**1b**), Pt­(dpyb-F)-C≡CNaph (**2b**), Pt­(dpyb)-C≡CPhen (**1c**) and Pt­(dpyb)-C≡CPhen (**2c**) were prepared as reported in the literature.^32,35^

#### Physical Measurements

NMR spectra were recorded in CDCl_3_ or in acetone-d_6_ at the Unitat de RMN of the Universitat de Barcelona with a Mercury 400 spectrometer (^1^H, 400 MHz; ^19^F, 376.5 MHz). Chemical shifts are given in δ values (ppm) relative to TMS (^1^H) or CFCl_3_ (^19^F) and coupling constants J are given in Hz. Infrared spectra were recorded in a Thermo Scientific FT-IR Nicolet iS5 spectrometer with an iD7 ATR accessory. Electrospray mass spectra were performed at the Unitat d’Espectrometria de Masses (Universitat de Barcelona) in a LC/MSD-TOF spectrometer using H_2_O–CH_3_CN 1:1 to introduce the sample.

#### Synthesis and Characterization

##### Synthesis of {Pt­(dpyb)}_2_(μ-1,3-(C≡C)_2_-Ph) (**5**)

{Pt­(dpyb)}_2_(μ-1,3-(C≡C)_2_-Ph) (**5**) was obtained by stirring a mixture of 0.003 g (0.027 mmol) of 1,3-diethynylbenzene and 0.001 g (0.027 mmol) of sodium hydroxide at room temperature under an atmosphere of nitrogen for 30 min. Afterward, 0.025 g (0.054 mmol) of compound Pt­(dpyb)­Cl (**1**) were added and the mixture was further stirred for 24 h. The obtained orange solid was filtered, washed with water, methanol and hexane and dried under Yield: 0.017 g (65%).

^**1**^ **H NMR** (CDCl_3_, 400 MHz): δ 9.47 [d, 4H, ^3^J­(Pt–H) = 47.2, ^3^J­(H–H) = 5.6, H^f^]; 7.93 [td, 4H, ^3^J­(H–H) = 7.8, ^4^J­(H–H) = 1.6, H^d^]; 7.72 [s, 1H, H^Di^]; 7.68 [d, 4H, ^3^J­(H–H) = 7.8, H^c^]; 7.52 [d, 2H, ^3^J­(H–H) = 8.4; H^Di^]; 7.52 [d, 4H, ^3^J­(H–H) = 7.8; H^b^]; 7.18–7.31 [m, 9H, H^a,e,Di^]. **MS-ESI** ^**+**^: *m*/*z* 977.15 [M + H]^+^ (calcd. 977.15). **IR**: υ 2060.12 (C≡C).

##### Synthesis of {Pt­(dpyb-F)}_2_(μ-1,3-(C≡C)_2_-Ph) (**6**)

{Pt­(dpyb-F)}_2_(μ-1,3-(C≡C)_2_-Ph) (**6**) was obtained as an orange solid by following the same method from 0.026 g (0.042 mmol) of compound [PtCl­{2,6-(C_5_H_4_N)_2_-4-FC_6_H_2_}] (**2**), 0.003 g (0.024 mmol) of 1,3-diethynylbenzene and 0.001 g (0.024 mmol) of sodium hydroxide. Yield: 0.013 g (54%).

^**1**^ **H NMR** (CDCl_3_, 400 MHz): δ 9.49 [dd, 4H, ^3^J­(Pt–H) = 48.0, ^3^J­(H–H) = 5.7, ^4^J­(H–H) = 1.6, H^f^]; 7.97 [td, 4H, ^3^J­(H–H) = 7.8, ^4^J­(H–H) = 1.6, H^d^]; 7.71 [s, 1H, H^Di^]; 7.65 [d, 4H, ^3^J­(H–H) = 7.8, H^c^]; 7.55 [d, 2H, ^3^J­(F–H) = 7.6, H^Di^]; 7.21–7.32 [m, 9H, H^b,e,Di^]. ^**19**^ **F NMR** (CDCl_3_, 376.5 MHz): δ −118.34 [t, 1F, ^3^J­(H–F) = 10.0]. **MS-ESI** ^**+**^: *m*/*z* 1013.13 [M + H]+ (calcd. 1013.13). **IR**: υ 2064.01 (C≡C).

### General Procedure for the Synthesis of Complexes **3x** and **4x**

To a solution of the corresponding precursor **3x** or **4x** in acetonitrile under an atmosphere of nitrogen, 0.5 or 1 equiv of silver triflate dissolved in acetonitrile were added. The mixture was stirred at room temperature covered from light for 1 h. Half of the solvent was removed and the obtained solid was filtered and dried under vacuum.

#### Synthesis of [{Pt­(dpyb)­(μ-C≡CThio)}_2_Ag]­[CF_3_SO_3_] (**3a**)

[{Pt­(dpyb)­(μ-C≡CThio)}_2_Ag]­[CF_3_SO_3_] (**3a**) was obtained as a red solid from 0.034 g (0.064 mmol) of Pt­(dpyb)-C≡CThio (**1a**) and 0.008 g (0.032 mmol) of silver triflate. Yield: 0.033 g (73%).

^**1**^ **H NMR** (acetone-*d* ^6^, 400 MHz): δ 9.17 [d, 4H, ^3^J­(Pt–H) = 47.2, ^3^J­(H–H) = 5.7, H^f^]; 8.01 [t, 4H, ^3^J­(H–H) = 7.6, H^d^]; 7.68 [d, 4H, ^3^J­(H–H) = 8.0, H^c^]; 7.50 [d, 4H, ^3^J­(H–H) = 7.7, H^b^]; 7.39 [d, 2H, ^3^J­(H–H) = 2.8, H^Thio^]; 7.26 [m, 4H, H^Thio^]; 7.22 [m, 6H, H^a,e^]. **MS-ESI** ^**+**^: *m*/*z* 1175.01 [M-CF_3_SO_3_]^+^ (calcd. 1175.01). **IR**: ν 2048.21 (C≡C).

#### Synthesis of [{Pt­(dpyb-F)­(μ-C≡CThio)}_2_Ag]­[CF_3_SO_3_] (**4a**)

[{Pt­(dpyb-F)­(μ-C≡CThio)}_2_Ag] [CF_3_SO_3_] (**4a**) was obtained as a red solid from 0.032 g (0.058 mmol) of Pt­(dpyb-F)-C≡CThio (**1a**) and 0.008 g (0.029 mmol) of silver triflate. Yield: 0.029 g (81%).

^**1**^ **H NMR** (acetone-*d* ^6^, 400 MHz): δ 9.16 [d, 4H, ^3^J­(Pt–H) = 48.1, ^3^J­(H–H) = 5.6, H^f^]; 8.03 [t, 4H, ^3^J­(H–H) = 7.8, H^d^]; 7.77 [d, 4H, ^3^J­(H–H) = 7.8, H^c^]; 7.38 [d, 2H, ^3^J­(H–H) = 2.6, H^Thio^]; 7.29 [d, 4H, ^3^J­(F–H) = 10.2, H^b^]; 7.25 [m, 4H, H^Thio^]; 7.21 [m, 4H, H^e^]. ^**19**^ **F NMR** (CDCl_3_, 376.5 MHz): δ −78.04 [s,3F, CF_3_SO_3_^–^]; −118.13 [t, 2F, ^3^J­(H–F) = 10.3]. **MS-ESI** ^**+**^: *m*/*z* 1211.01 [M-CF_3_SO_3_]^+^ (calcd. 1210.99). **IR**: ν 2054.68 (C≡C).

#### Synthesis of [{Pt­(dpyb)­(μ-C≡CNaph)}_2_Ag]­[CF_3_SO_3_] (**3b**)

[{Pt­(dpyb)­(μ-C≡CNaph)}_2_Ag]­[CF_3_SO_3_] (**3b**) was obtained as a red solid from 0.048 g (0.084 mmol) of Pt­(dpyb)-C≡CNaph (**1b**) and 0.011 g (0.042 mmol) of silver triflate. Yield: 0.042 g (69%).

^**1**^ **H NMR** (acetone-*d* ^6^, 400 MHz): δ 9.20 [d, 4H, ^3^J­(Pt–H) = 47.6, ^3^J­(H–H) = 5.6, H^f^]; 8.21 [s, 2H, H^Naph^]; 7.98 [t, 4H, ^3^J­(H–H) = 7.7, H^d^], 7.79 [m, 6H, H^Naph^]; 7.67–7.72 [m, 6H, H^c,Naph^], 7.56 [d, 4H, ^3^J­(H–H) = 7.7, H^b^]; 7.46 [m, 4H, H^Naph^]; 7.23 [m, 6H, H^a,e^]. **MS-ESI** ^**+**^: *m*/*z* 1263.13 [M-CF_3_SO_3_]^+^ (calcd. 1262.13). **IR**: ν 2054.98 (C≡C).

#### Synthesis of [{Pt­(dpyb-F)­(μ-C≡CNaph)}_2_Ag]­[CF_3_SO_3_] (**4b**)

[{Pt­(dpyb-F)­(μ-C≡CNaph)}_2_Ag]­[CF_3_SO_3_] (**4b**) was obtained as a red solid from 0.042 g (0.070 mmol) of Pt­(dpyb-F)-C≡CNaph (**2b**) and 0.009 g (0.035 mmol) of silver triflate. Yield: 0.039 g (75%).

^**1**^ **H NMR** (acetone-*d* ^6^, 400 MHz): δ 9.23 [d, 4H, ^3^J­(Pt–H) = 47.8, ^3^J­(H–H) = 5.7, H^f^]; 8.23 [s, 2H, H^Naph^]; 8.04 [t, 4H, ^3^J­(H–H) = 7.8, H^d^], 7.81 [m, 6H, H^Naph^]; 7.72 [m, 6H, H^c,Naph^]; 7.44 [m, 4H, H^Naph^]; 7.27 [d, 4H, ^3^J­(F–H) = 10.1, H^b^]; 7.24 [m, 4H, H^e^]. ^**19**^ **F NMR** (CDCl_3_, 376.5 MHz): δ −77.97 [s,3F, CF_3_SO_3_^–^]; −118.48 [t, 2F, ^3^J­(H–F) = 10.3]. **MS-ESI** ^**+**^: *m*/*z* 1299.11 [M-CF_3_SO_3_]^+^ (calcd. 1299.11). **IR**: ν 2059.35 (C≡C).

#### Synthesis of [{Pt­(dpyb)­(μ-C≡CPhen)}_2_Ag]­[CF_3_SO_3_] (**3c**)

[{Pt­(dpyb)­(μ-C≡CPhen)}_2_Ag]­[CF_3_SO_3_] (**3c**) was obtained as a red solid from 0.043 g (0.070 mmol) of Pt­(dpyb)-C≡CPhen (**1c**) and 0.009 g (0.034 mmol) of silver triflate. Yield: 0.041 g (78%).

^**1**^ **H NMR** (acetone-*d* ^6^, 400 MHz): δ 9.27 [d, 4H, ^3^J­(Pt–H) = 48.0, ^3^J­(H–H) = 5.7, H^f^]; 8.98 [d, 2H, ^3^J­(H–H) = 7.8, H^Phen^]; 8.81 [d, 2H, ^3^J­(H–H) = 7.8, H^Phen^]; 8.69 [d, 2H, ^3^J­(H–H) = 7.9, H^Phen^]; 8.08 [s, 2H, H^Phen^]; 8.07 [t, 4H, ^3^J­(H–H) = 7.8, H^d^], 7.79 [d, 4H, ^3^J­(H–H) = 7.8, H^c^]; 7.75 [m, 2H, H^Phen^], 7.66 [m, 4H, H^Phen^]; 7.56 [m, 4H, H^Phen^]; 7.32 [d, 4H, ^3^J­(H–H) = 7.7, H^b^]; 7.19–7.22 [m, 6H, H^a,e^]. **MS-ESI** ^**+**^: *m*/*z* 1362.14 [M-CF_3_SO_3_]^+^ (calcd. 1363.16). **IR**: ν 2045.22 (C≡C).

#### Synthesis of [{Pt­(dpyb-F)­(μ-C≡CPhen)}_2_Ag]­[CF_3_SO_3_] (**4c**)

[{Pt­(dpyb-F)­(μ-C≡CPhen)}_2_Ag]­[CF_3_SO_3_] (**4c**) was obtained as a red solid from 0.034 g (0.052 mmol) of compound Pt­(dpyb-F)-C≡CPhen (2**c**) and 0.007 g (0.026 mmol) of silver triflate. Yield: 0.034 g (83%).

^**1**^ **H NMR** (acetone-*d* ^6^, 400 MHz): δ 9.25 [d, 4H, ^3^J­(Pt–H) = 48.4, ^3^J­(H–H) = 5.7, H^f^]; 8.88 [d, 2H,^3^J­(H–H) = 7.8, H^Phen^]; 8.71 [d, 2H, ^3^J­(H–H) = 7.8, H^Phen^]; 8.67 [d, 2H, ^3^J­(H–H) = 7.7, H^Phen^]; 8.10 [s, 2H, H^Phen^]; 8.03 [t, 4H, ^3^J­(H–H) = 7.8, H^d^], 7.81 [d, 4H, ^3^J­(H–H) = 7.8, H^c^]; 7.73 [m, 2H, H^Phen^]; 7.63 [m, 4H, H^Phen^]; 7.51 [m, 4H, H^Phen^]; 7.32 [d, 4H, ^3^J­(F–H) = 10.0, H^b^]; 7.18 [m, 4H, H^e^]. ^**19**^ **F NMR** (CDCl_3_, 376.5 MHz): δ −77.91 [s,3F, CF_3_SO_3_^–^]; −118.09 [t, 2F, ^3^J­(H–F) = 10.3]. **MS-ESI** ^**+**^: *m*/*z* 1399.16 [M-CF_3_SO_3_]^+^ (calcd. 1399.14) **IR**: ν 2043.84 (C≡C).

#### Synthesis of [{Pt­(dpyb))_2_(μ-1,3-(C≡C)_2_-Ph)}_2_Ag_2_]­[CF_3_SO_3_]_2_ (**7**)

[{Pt­(dpyb))_2_(μ-1,3-(C≡C)_2_-Ph)}_2_Ag_2_]­[CF_3_SO_3_]_2_ (**7**) was obtained as a red solid from 0.054 g (0.055 mmol) of compound {Pt­(dpyb)}_2_(μ-1,3-(C≡C)_2_-Ph) (**5**) and 0.014 g (0.055 mmol) of silver triflate. Yield: 0.076 g (56%).

^**1**^ **H NMR** (acetone-*d* ^6^, 400 MHz): δ 9.11 [d, 8H, ^3^J­(Pt–H) = 47.4, ^3^J­(H–H) = 5.6, H^f^]; 8.00 [t, 8H, ^3^J­(H–H) = 7.8, H^d^]; 7.96 [s, 2H, H^Di^]; 7.85 [d, 8H, ^3^J­(H–H) = 7.8, H^c^]; 7.49 [d, 4H, ^3^J­(H–H) = 8.4; H^Di^]; 7.54 [d, 8H, ^3^J­(H–H) = 7.8; H^b^]; 7.28 [s, 2H, H^Di^]; 7.22 [m, 12H, H^a,e^]. **MS-ESI** ^**+**^: *m*/*z* 1211.10 [M-2CF_3_SO_3_+CF_3_SO_3_Ag]^2+^ (calcd. 1211.98).

^+^. **IR**: υ 2034.19 (C≡C).

#### Synthesis of [{Pt­(dpyb-F))_2_(μ-1,3-(C≡C)_2_-Ph)}_2_Ag_2_]­[CF_3_SO_3_]_2_ (**8**)

[{Pt­(dpyb-F))_2_(μ-1,3-(C≡C)_2_-Ph)}_2_Ag_2_]­[CF_3_SO_3_]_2_ (**8**) was obtained as a red solid from 0.040 g (0.040 mmol) of compound {Pt­(dpyb-F)}_2_(μ-1,3-(C≡C)_2_-Ph) (**6**) and 0.010 g (0.040 mmol) of silver triflate. Yield: 0.059 g (58%).

^**1**^ **H NMR** (acetone-*d* ^6^, 400 MHz): δ 9.13 [d, 8H, ^3^J­(Pt–H) = 48.2, ^3^J­(H–H) = 5.6, H^f^]; 8.03 [t, 8H, ^3^J­(H–H) = 7.8, H^d^]; 7.92 [s, 2H, H^Di^]; 7.80 [d, 8H, ^3^J­(H–H) = 7.7, H^c^]; 7.51 [d, 2H, ^3^J­(F–H) = 7.6, H^Di^]; 7.31 [s, 2H, H^Di^]; 7.27 [d, 8H, ^3^J­(F–H) = 10.1, H^b^]; 7.19 [m, 8H, H^e^]. ^**19**^ **F NMR** (CDCl_3_, 376.5 MHz): δ −77.83 [s,3F, CF_3_SO_3_^–^]; −118.06 [t, 4F, ^3^J­(H–F) = 10.3]. **MS-ESI** ^**+**^: *m*/*z* 1120.87 [M-2CF_3_SO_3_+F^–^]^2+^ (calcd. 1247.97).^+^. **IR**: ν 2039.84 (C≡C).

### Photophysical Studies

Absorption spectra of 10^–5^ M acetonitrile solutions of the final compounds were recorded with a Cary 100 scan 388 Varian UV spectrometer at 298 K. Emission spectra of solutions and powders were recorded using a QePro compact spectrometer (Ocean Optics). Excitation spectra in solution were recorded with a Fluorolog fluorescence spectrometer (Jobin Yvon). Time-resolved decays in solution and powder were recorded with a Horiba DeltaFlex TCSPC system using a 330 nm SpectraLED light source. Temperature-dependent experiments were conducted using a liquid nitrogen cryostat VNF-100 (sample in flowing vapor, Janis Research) under nitrogen atmosphere, while measurements at room temperature were recorded under vacuum in the same cryostat. Solutions were degassed using five freeze–pump–thaw cycles. Emission quantum yields were determined with a Hamamatsu Quantaurus QY absolute photoluminescence quantum yield spectrometer C11347. Time-resolved spectra were recorded using a home-built transient photoluminescence setup comprising of an EKSPLA Nd:YAG laser with a third harmonic emitting at 355 nm, a Horiba TRIAX spectrograph and an iCCD camera 4Picos from Stanford Computer Optics. Details of the setup are provided in an earlier publication.^36^

### Calculations

We use density functional theory (DFT) and time-dependent DFT (TD-DFT) implemented in Orca 5.0.3 ^37,38^ in order to gain an additional insight into the luminescence mechanisms of the studied molecules. Ground state (S_0_) and triplet (T_1_) excited state geometries were optimized at the BP86 ^39^/def2-SVP^40^/CPCM­(acetonitrile) level of theory which was found to be the most optimal for this task. All molecular orbital (MO) iso surfaces were visualized using Gabedit 2.5.0.^41^ Geometries were generally verified to be true energy minima by a frequency calculation. We have observed that in some cases very small negative frequencies were obtained, attributed to numerical errors, and suggesting that the affected structures show geometries close to minimal. All optimizations were performed with tight self-consistent field (SCF) and geometry convergence criteria. Single point energy of TD-DFT states was calculated at the T_1_ geometry using the B3LYP/def2-SVP/CPCM­(acetonitrile) level of theory.

### Fabrication and Characterization of Light-Emitting Electrochemical Cells (LEECs)

LEECs were fabricated by spin-coating with the top Al electrode fabricated using vacuum thermal evaporation. Precleaned indium–tin-oxide (ITO) coated glass substrates with a sheet resistance of 20 Ω/sq and ITO thickness of 100 nm were used. The substrates were first cleaned with water, acetone and then sonicated in acetone, and isopropanol, for 15 min each time. Substrates were dried with compressed air and transferred into an oxygen plasma generator for 6 min at full power. Thermally deposited Al electrode was produced using Kurt J. Lesker Spectros II deposition system at 10^–6^ mbar base pressure. The electrode was deposited at a rate of 1 Å s^–1^. Characterization of LEEC devices was conducted in a 10-in. integrating sphere (Labsphere) connected to a Source Measure Unit (SMU, Keithley) and coupled with a compact spectrometer USB4000 (Ocean Optics). Further details are available in reference.^42^ Devices of 4 × 2 mm pixel size were fabricated.

The LEEC structure comprised PEDOT:PSS Al4083 (Ossila) as the hole injection layer, Al cathode, and an emissive layer composed of PVK {poly­(9-vinylcarbazole), *M* = 90 000 Da, Sigma-Aldrich}, OXD7 {1,3-bis­[2-(4-*tert*-butylphenyl)-1,3,4-oxadiazo-5-yl]­benzene, Lumtec}, and THABF_4_ (Sigma-Aldrich). The Al4083 layer was obtained by dynamic spin-coating method at 4000 rpm with the film annealed at 120 °C for 15 min. The emissive layer was obtained with static spin-coating method at 1000 rpm from a 10 mg mL^–1^ (PVK+OXD7+THABF_4_+emitter) solution in CH_2_Cl_2_ and dried at high vacuum without annealing.

### Cyclic Voltammetry

Cyclic voltammetry was conducted in a three-electrode, one-compartment cell. All measurements were performed using 0.1 M Bu_4_NBF_4_ (99%, Sigma-Aldrich, dried) solution in dichloromethane (ExtraDry AcroSeal, Acros Organics). All solutions were purged with nitrogen prior to measurement and the measurement was conducted in a nitrogen atmosphere. Electrodes used in the experiment were: working (Pt disc d = 1 mm), counter (Pt wire), reference (Ag/AgCl calibrated against ferrocene). All cyclic voltammetry measurements were performed at room temperature with a scan rate of 50 mV s^–1^.

The ionization potential (IP) and electron affinity (EA) are obtained from onset redox potentials; these figures correspond to HOMO and LUMO values, respectively. The ionization potential is calculated from onset oxidation potential IP = *E*_ox_^CV^ + 5.1 and the electron affinity is calculated from onset reduction potential EA = E_red_^CV^ + 5.1.^43–46^ An uncertainty of ± 0.02 V is assumed for the electrochemical onset potentials.

## Results and Discussion

### Synthesis and Characterization

Complexes **Pt­(dpyb)-C≡CR**_**2**_ (**1x**) and **Pt­(dpyb-F)-C≡CR**_**2**_ (**2x**), where dpyb is 2,6-dipyridylbenzene, with R_1_ = H, while {dpyb-F}-H is 4-fluoro-2,6-dipyridylbenzene, with R_1_ = F, and x = a for R_2_ = thiophene; b for R_2_ = naphthalene and c for R_2_ = phenantrene, are depicted in Scheme 1. The complexes were prepared following the procedure previously reported by us.^32^ Subsequently, they were treated with silver triflate in a 2:1 stoichiometry in acetonitrile to yield heteronuclear complexes **3x** and **4x**, respectively. The resultant precipitate was stirred for 24 h at RT followed by washing with water, methanol, and hexane.

**Scheme 1:**
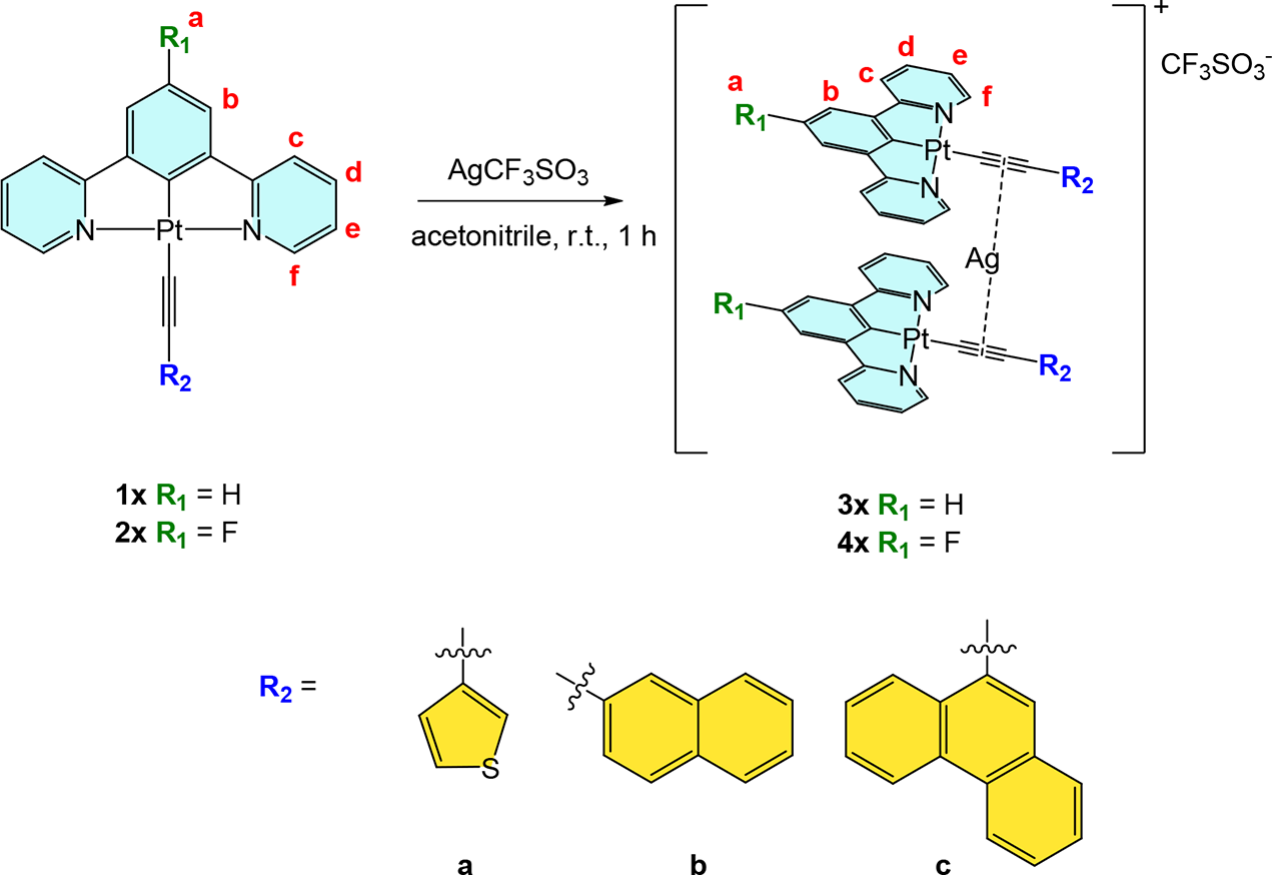
Synthetic Procedure for the Pt­(II)–Ag­(I) Heterometallic Ionic Complexes **3x** and **4x** Starting from **1x** and **2x**

We determined the Pt:Ag complex stoichiometry to be 2:1 using fluorometric titration by addition of AgOTf in 0.1 equiv to a 5 × 10^–5^ M solution of the Pt compound **1x** or **2x** in acetonitrile. We observe that upon addition of Ag­(I) ions, a new broad band at λ_PL_ ∼ 600–700 nm emerges and is likely associated with a ^3^MMLCT state. The analysis of the relationship between the amount of the added AgOTf and the intensity of the said ^3^MMLCT luminescent band points at the Ag­(I) ion binding two acetylene groups together (see Figures S11 and S12 in the SI). At this point we speculate that the long wavelength photoluminescence observed upon formation of the heteronuclear complexes originates from Pt···Pt short contacts giving rise to ^3^MMLCT excited states. Given the experimental Ag–C bond length in such complexes at ∼2.2 Å^47^ it is unlikely, however, that these ^3^MMLCT states originate from intramolecular interactions as the typical Pt···Pt distance allowing formation of the said states is ∼3 Å.^48,49^ We study this behavior in more detail later in the text.

We have also obtained two new dinuclear cyclometalated Pt­(II) complexes containing 1,3-diethynylbenzene as a bridging ditopic ligand. In this case the two acetylide groups coordinate to two different Pt­(II) centers when reacted with 2 equiv of the previously reported chloride parent complexes **Pt­(dpyb)-Cl** (**1**)^35^ and **Pt­(dpyb-F)-Cl** (**2**)^50^ in the presence of sodium hydroxide as a base (Scheme 2). The final compounds **{Pt­(dpyb)}**_**2**_ **(μ-1,3-(C≡C)**_**2**_ **-Ph)** (**5**) and **{Pt­(dpyb-F)}**_**2**_ **(μ-1,3-(C≡C)**_**2**_ **-Ph)** (**6**) precipitated as orange solids and were washed with water, methanol, and hexane.

**Scheme 2:**
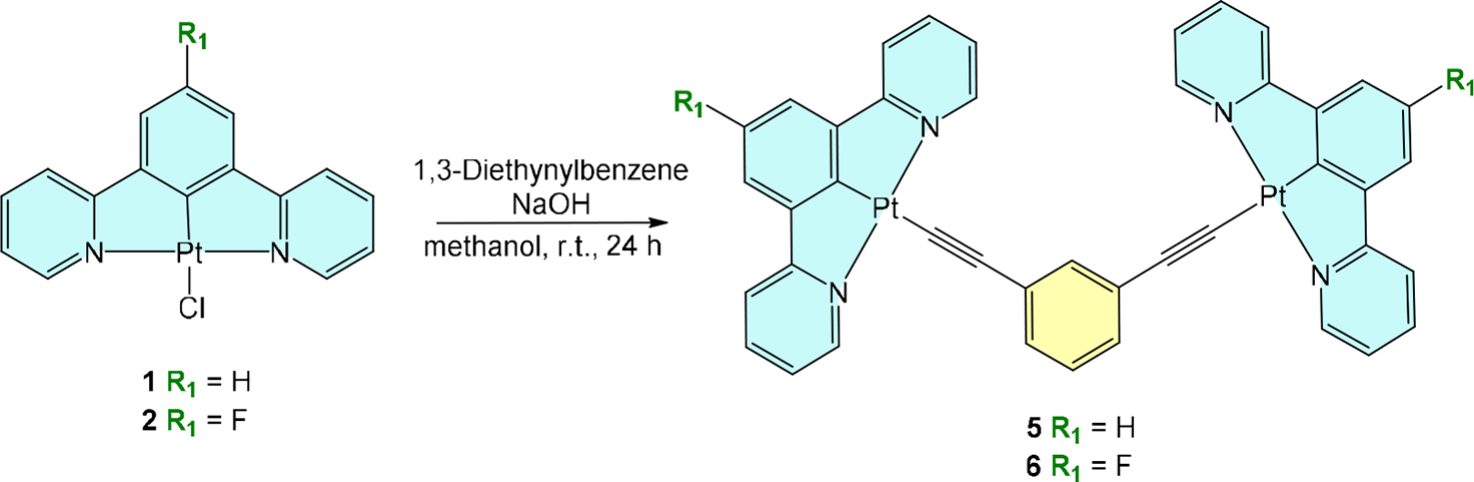
Synthesis of Dinuclear Pt­(II) Complexes **5** and **6**

^1^H NMR spectra of both complexes show protons of the two equivalent dpyb/dpyb-F units, while the alkynyl protons are absent. The formation of the desired products is also supported by a significant shift of the protons that belong to the cyclometalated unit, such as a ca. 0.10–0.25 ppm downfield shift in the pyridine proton in *ortho* position to the heteroatom compared to that of the **Pt­(dpyb)-Cl** precursors. Additionally, compound **6** displays a triplet in the ^19^F NMR spectrum, associated with the F- in the dpyb-F ligand.

Infrared spectroscopy shows the C≡C stretching vibration (∼2070 cm^–1^) for both compounds along with the absence of the band assigned to the stretching of the terminal **H-**C≡C protons (∼3300 cm^–1^). Identity of complexes **5** and **6** is further confirmed with mass spectroscopy, which reveals expected [M + H]^+^ molecular ions (Figures S7 and S8).

Titration of complexes **5** and **6** with AgOTf gives analogous results to those observed for **3x** and **4x** with the Ag^+^ ion binding two C≡C units each, but as there are two C≡C units per molecule then there is effectively one Ag^+^ ion per molecule in the resultant complexes **7** and **8**, rather than 0.5 as in complexes **3x** and **4x** (Figure S13). It can be expected that the Ag^+^ ion binds the two C≡C moieties through η^2^ binding motifs.^51,52^ Alternative binding modes do exist with simultaneous coordination to the alkynyl and to the platinum metal center^53,54^ (Scheme 3).

**Scheme 3:**
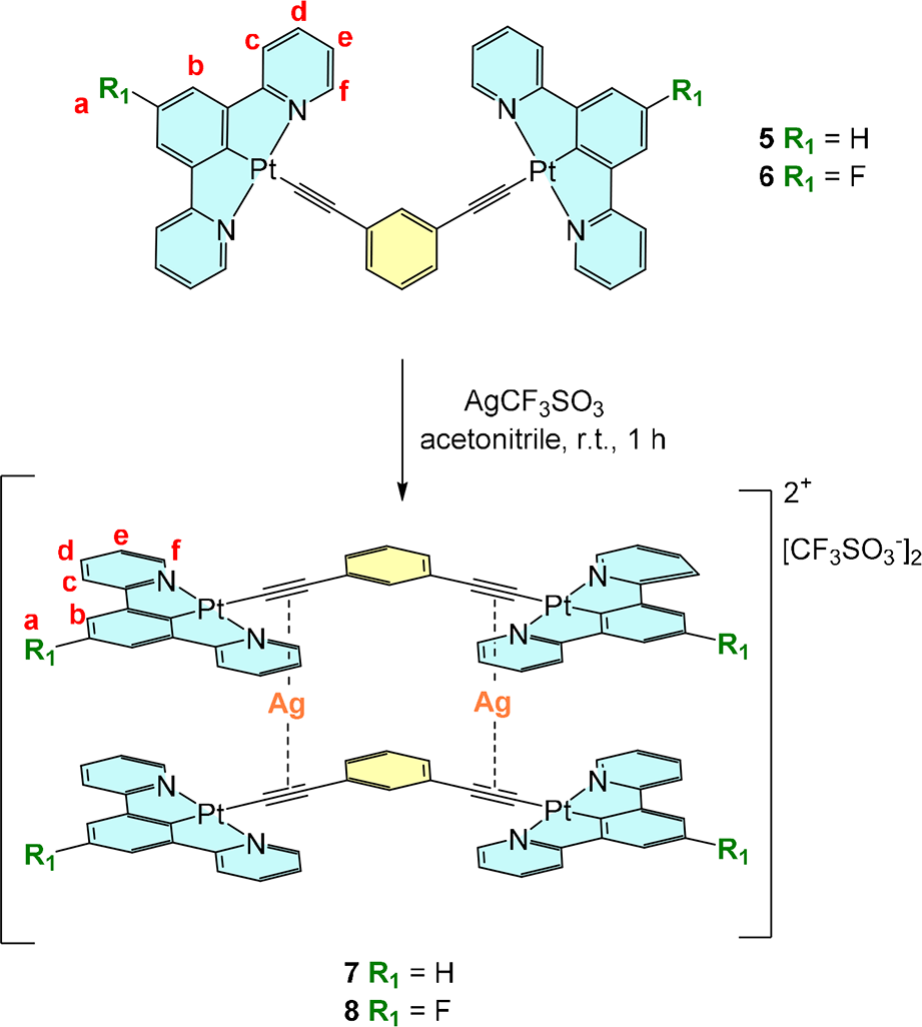
Synthesis and Proposed Structures of the Pt­(II)/Ag­(I) Heterometallic Complexes **7** and **8**

As expected, ^1^H NMR spectra of complexes **3x**, **4x**, **7**, and **8** display signals of all the protons present in the parent mononuclear complexes but shifted upfield in respect to the parent molecules. In particular, a larger shift is present for the protons of the *NCN* dpyb/dpyb-F ligands, with the most significant being the proton *ortho* to the heteroatom (H^f^), shifted by *ca*. 0.4 ppm. ^19^F NMR reveals a singlet around −78 ppm assigned to the CF_3_ group of the triflate ion. Compounds **4x** and **8** present an additional triplet around −118 ppm which corresponds to the fluorine atom in the dpyb-F *NCN* ligand. This signal is not significantly shifted when compared to the ^19^F NMR of their corresponding precursors. Integrals of the two ^19^F in complexes containing the dpyb-F ligand agree with the expected ionic composition of the heteronuclear complexes.

We used infrared spectroscopy to probe the effects of Ag^+^ ions binding to the C≡C units. The C≡C stretching vibration is shifted to lower frequencies in **3x** and **4x** as compared to the **1x** and **2x** precursors, with a decrease of around 30 cm^–1^ in each case,^32^ in agreement with the behavior observed in analogous complexes of d^10^ metal ions. A similar effect is observed in **7** and **8** vs **5** and **6**. Additionally, the [M-CF_3_SO_3_]^+^ or [M-(2CF_3_SO_3_+F)]^+^ ions are found in mass spectrometry data, further confirming the identity of the desired products (Figures S1–S6, S9, and S10).

### DFT Calculations

Density functional theory (DFT) and time-dependent DFT (TD-DTF) implemented in the Orca 5.0.3 software package^37,38^ were used to study the electronic excited states of our heteronuclear complexes. Ground (S_0_) and triplet excited state (T_1_) geometries of the studied complexes were obtained at the BP86 ^39^/def2-svp^40^ level of theory using the CPCM solvent model for acetonitrile. Single point energy calculations used the B3LYP/def2-svp,^40^ level of theory and identical settings for the CPCM solvent model. The structure of the studied complexes was simplified to facilitate computations: (1) we first optimized structure of a silver­(I) diacetylene complex considering the η^2^ Ag–C binding and established Ag–C bond lengths at 2.15 Å (in agreement with the experimental bond lengths of ∼2.2 Å^47^), these bond lengths were then constrained for geometry optimizations of the heteronuclear complexes; (2) the structure of **7** and **8** was simplified to contain only one Pt­(*NCN*) unit, this simplification is generally appropriate considering the photophysical behavior of these complexes described in the later part of this work. We use **7′** and **8**′**** to denote these model molecules, as they structurally differ from **7** and **8**. The structures represent the first of the two potentially possible isomeric forms: head-to-head and head-to-tail. The complexes were modeled as possessing a single positive charge and the counterions are omitted. Based on our results, we believe that the two hypothetical isomeric forms display similar photophysical characteristics, hence analysis of both forms is unnecessary. The results are summarized in Figures 1–3, and S14–S22 as well as Tables S2–S9.

**Figure 1:**
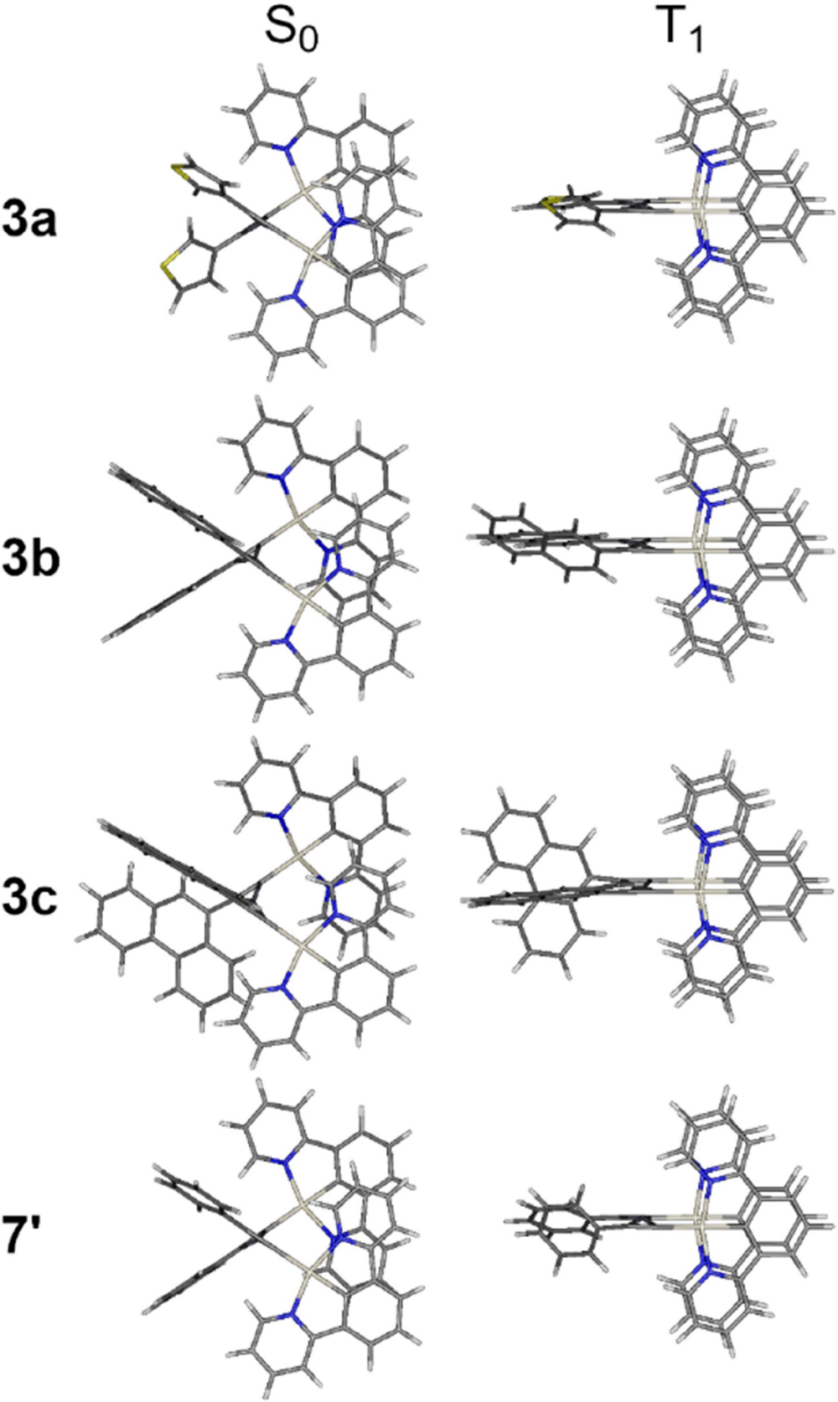
Ground state (S_0_) and triplet excited state (T_1_) geometries of complexes **3a**–**3c** and model complex **7**′****.

The ground state (S_0_) geometries of our complexes significantly differ from those of the triplet excited state (T_1_). At ground state the two Pt­(*NCN*) units experience Coulombic repulsion, forming a scissor-like geometry (Figure 1). At the T_1_ geometry the Pt­(*NCN*) units attract each other as observed computationally in other cases,^48,55^ leading to a defined head-to-head orientation. There are clearly no Pt···Pt contacts in the S_0_ geometry, while the intermetallic distance at T_1_ geometry exceeds ∼3 Å, which prohibits interaction of the d_*z*_^2^ orbitals of the two metal centers.^56^ The aryl groups of the Ar–C≡C moiety generally orient themselves rather orthogonally to the Pt­(*NCN*) plane, except in **3c**/**4c** where the two phenanthrene units display sufficient electronic repulsion to prevent this type of arrangement. In this latter case one of the phenanthrenes is rather orthogonal, while the other more planar with the Pt­(*NCN*) unit, hence making them inequivalent.

We observe that the aryl group attached to the distal end of the acetylide auxiliary ligand is not entirely inert and may significantly influence the photophysical properties of these complexes. For example, fused aromatic systems often display relatively low-lying local triplet excited states which may be of similar or lower energy to the energy of the T_1_ state associated with the cyclometalated Pt­(*NCN*) unit. In order to verify this view, we studied the molecular orbital iso surfaces representing the dominant transitions involved in the S_1_–S_3_ and T_1_-T_3_ states. These are presented in Figures 2 and 3 for **3a**–**3c** and **7**′****, while supplementary figures pertaining to complexes **4a**–**4c** and **8**′**** as well as tabulated excited state characteristics are presented in the SI, Figures S15–S22 and Tables S2–S9.

**Figure 2:**
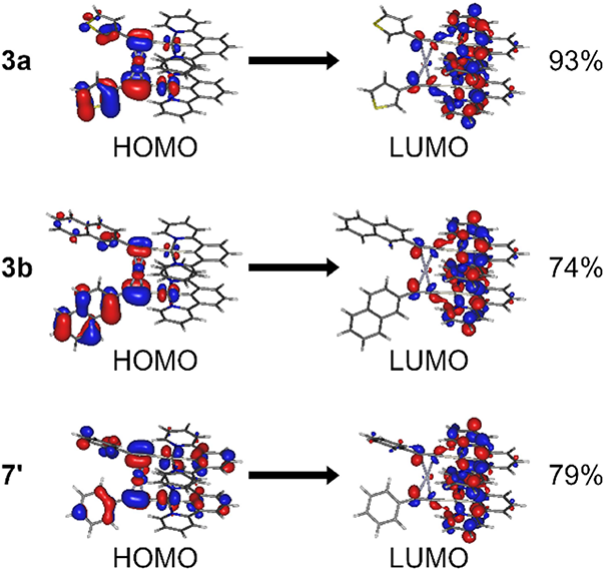
Dominant electronic transitions associated with the triplet excited state (T_1_) in complexes **3a**, **3b** and model complex **7**′**** at their respective T_1_ geometry. Percent values denote the *c* ^2^ normalized to 100% where *c*-coefficient of the given orbital pair to the electron transition.

The general picture is similar throughout the whole series: Pt­(II) d orbitals (except d_*z*_^2^) alongside Ag­(I) d orbitals and Ar–C≡C π orbitals (including π orbitals of the Ar fragment) contribute to the HOMO, while the *NCN*-coordinating ligand contributes to LUMO. This orbital pairing gives a general MXLCT (metal-X-to-ligand charge-transfer, where X = −C≡C–Ar in this work) character to the T_1_ excited state. In other words: all complexes generally share the excited state characteristics of the related Pt­(*NCN*)-X group of complexes in their monomeric forms. Here the Ag­(I) ion, although contributing to the HOMO, does not appear to significantly alter molecular orbital energy, which is somewhat consistent with the ancillary ligand X generally not visibly affecting the photophysics in this group of complexes (perhaps except X = SPh).^56,57^ This general picture is common to complexes **3a**/**4a** as well as **7**′****/**8**′****.

In complexes **3b**/**4b** and **3c**/**4c** the effect of the aromatic group Ar at the far end of the acetylide ligand cannot be neglected. Here we focus on **3c** and generalize the discussion over the other three complexes. In **3c** the T_1_ state displays a mixed MXLCT + LC (ligand-centered) + LLCT (ligand–ligand or interligand CT) character, where the LC component originates from the phenanthrene aromatic group. The LC component is mainly due to the HOMO → LUMO + 5 transition (Figures 3 and 4). In this case the mixed character of the state may result in spectral characteristics of the T_1_ emission being influenced by the vibronic levels of phenanthrene, with the Pt­(II) center providing sufficient spin–orbit coupling to allow for a relatively short-lived photoluminescence. A similar MXLCT + LC character is also observed for the T_2_ and T_3_ excited states, with the LC character being due to the same HOMO→LUMO+5 transition. **4c** presents a similar orbital picture, with a pronounced contribution of the LC (phenanthrene) character.

**Figure 3:**
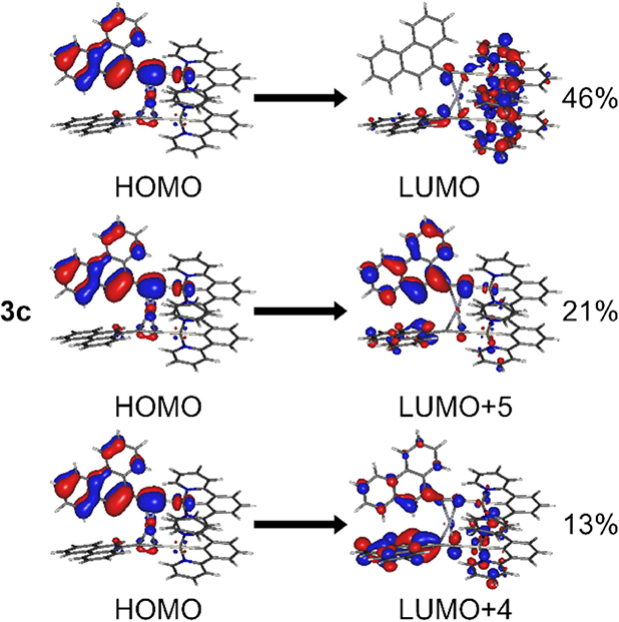
Dominant electronic transitions associated with the triplet excited state (T_1_) in complex **3c** at its T_1_ geometry. Percent values denote the *c* ^2^ normalized to 100% where c–coefficient of the given orbital pair to the electron transition.

**Figure 4:**
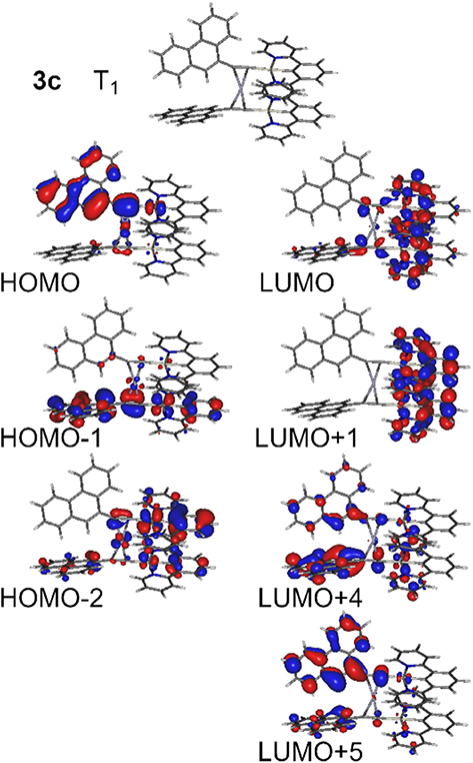
Molecular geometry at the T_1_ state of complex **3c** and the molecular orbitals relevant to the three lowest singlet and triplet excited states.

In **3b** we observe a somewhat similar behavior to that in **3c**/**4c**, but the T_1_ state displays an MXLCT character, while it is the T_2_ that displays an LC admixture. In **3b** the T_2_ is 57 meV above the T_1_ (Table S3). As these states are near-degenerate in the calculation, it is possible they are similarly nearly degenerate in the real system and hence in a thermal equilibrium. Alternatively, due to under or overestimation of the solvent effects the MXLCT + LC state in the experimental system may lay slightly below that of the MXLCT state. Hence, a similar influence of the naphthalene aromatic unit to that of the phenanthrene unit in **3c**/**4c** is likely to be present in **3b**. In **4b** the MXLCT + LC is T_3_, located 154 meV above the T_1_. In this case it is unlikely for the LC character to be present in phosphorescence of **4b**. As shown later in the text, the behavior hypothesized above is confirmed for both **3b** and **4b** as well as **3c** and **4c**.

### Steady State Absorption and Photoluminescence Spectra

Absorption and emission spectra of all complexes were recorded in dilute (*c* = 10^–5^ M) acetonitrile solutions at room temperature. The solutions for photoluminescence studies were degassed using a pump-freeze procedure described earlier.^36^ The results are summarized in Table 1.

**Table 1:** Spectral Data for All Complexes in Dilute Acetonitrile Solutions (Degassed for PL Measurements) at 298 K

compound	absorption λ_abs_/nm (ε/10^3^M^–1^cm^–1^)	emission λ_em_/nm
**3a**	287 (38.6), 311 (18.3), 332 (15.5), 357 (12.2), 374 (13.8), 424sh (2.0)^a^	486, 514, 653^b^
**3b**	287 (35.5), 307 (28.6), 383 (9.4), 427sh (4.8)^a^	487, 514, 674^b^
**3c**	287 (34.4), 312 (28.6), 332 (18.7), 375 (10.6), 424sh (3.5)^a^	486, 515, 645^b^
**7**	287 (102.5), 333 (42.0), 371 (31.4), 423sh (10.0)^a^	484, 514, 647^b^
**4a**	286 (15.9), 386 (5.3), 427sh (3.5)^a^	519, 675sh^a^ ^,^ ^b^
**4b**	286 (32.8), 310 (19.0), 330 (17.5), 354 (15.0), 391 (7.1), 410sh (3.8)^a^	495, 523
**4c**	288 (54.9), 312 (41.8), 329 (35.8), 394 (16.2), 432sh (9.6)^a^	499, 540, 675^b^
**8**	286 (109.0), 323 (40.6), 385 (33.0), 425sh (12.8)^a^	521, 648^b^

a Shoulder.

b ^3^MMLCT emission.

Compounds **3x** and **4x** present several absorption bands, with the spectra generally matching those of their parent mono-Pt­(II) complexes but with the presence of a broader shoulder at around 400–430 nm, which we believe originates from the interaction of the Ag^+^ ion coordinated to the C≡C units (Figure 5). The higher energy absorption bands can be assigned to ^1^π–π* transitions intrinsic to the cyclometalated Pt­(*NCN*) units.^28^ In the lower energy region, less intense bands are observed that may correspond to mixed charge transfer and ligand centered transitions.^32,58,59^ Complexes **7** and **8** present absorption coefficients that are visibly larger than those of the rest of the complexes. This is consistent with their molecular structure as they contain duplicated chromophoric units.

**Figure 5:**
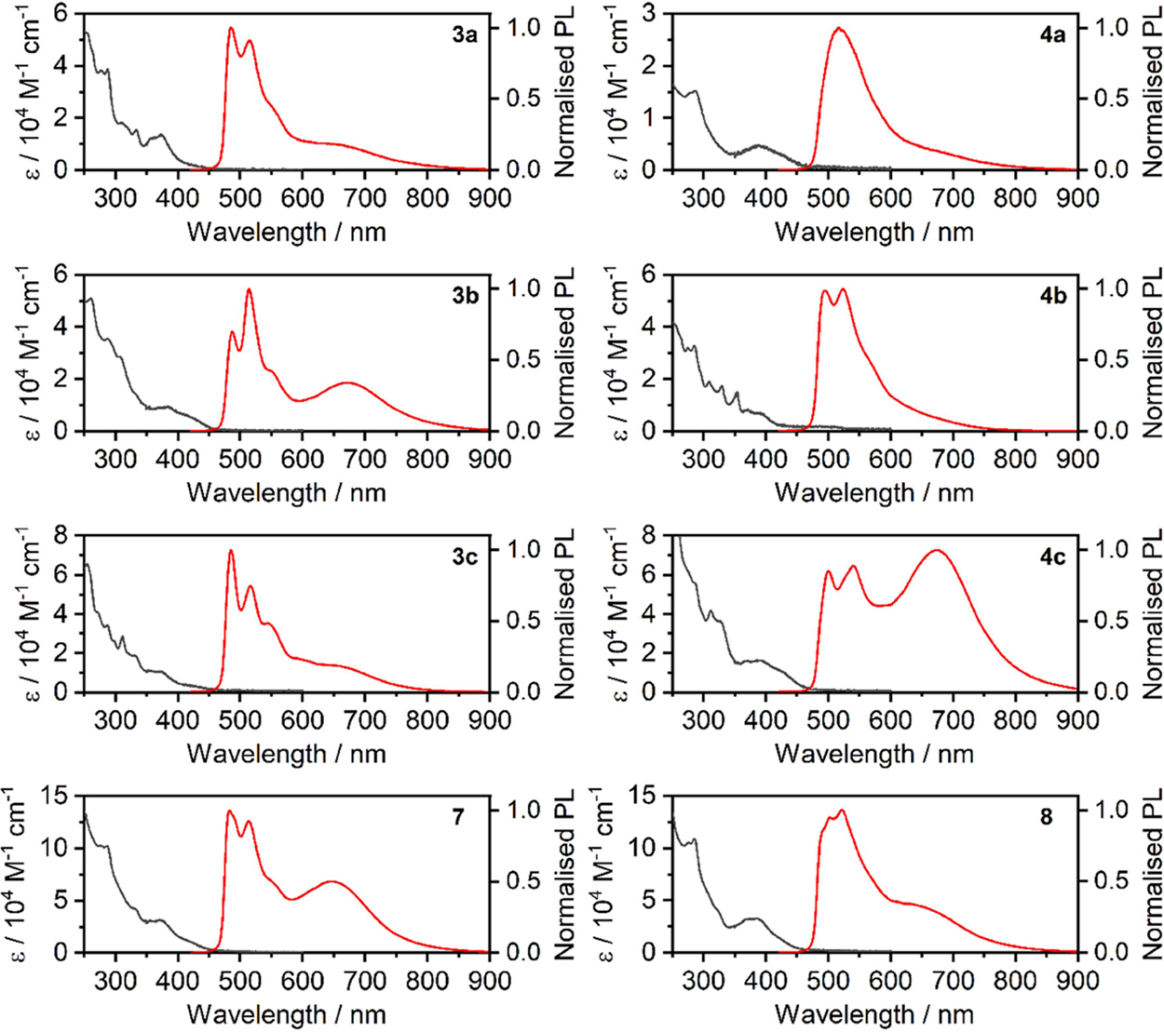
Absorption and photoluminescence spectra of complexes **3x**, **4x**, **7**, and **8** in dilute, *c* = 10^–5^ M acetonitrile solutions at room temperature.

Emission spectra of complexes **3x**, **4x**, **7**, and **8** display two main luminescent components (Figure 5). The high energy component displays several (presumably vibronic) bands in the 450–600 nm spectral region–some of them are typical of the mixed ^3^MXLCT PL of the parent **Pt­(dpyb)-Cl** and related complexes.^32,60,61^ However, we note that the aromatic group connected to the ancillary acetylide has a profound effect on the PL spectrum in a diluted solution. The low energy emission spans the 600–800 nm spectral region and corresponds to the ^3^MMLCT state arising from short contacts formed by two interacting Pt­(II) centers.^48,62^ This effect is more pronounced in the heterometallic complexes, being observed even at low concentrations, probably due to higher rigidity compared to the homometallic precursors **1** *x* **/2x**.

However, the relative intensity of this ^3^MMLCT band is highly influenced by the nature of the aromatic unit attached to the acetylide ligand–the contribution of this band being negligible in **4a** and **4b**. Our assessment suggests that the two Pt­(II)-containing units bound with the Ag­(I) ion are at a distance exceeding the typical value of ∼3 Å, hence the ^3^MMLCT should originate from *inter*molecular interactions–in agreement with our calculations and earlier works.^56^ In other words, the long wavelength ^3^MMLCT PL likely originates from intermolecular aggregates or excimers rather than from intramolecular interactions.

Φ_PL_ values are rather moderate for all complexes and slightly lower in **3x** and **4x** than their monometallic parent complexes **1x** and **2x** ^28^ (Table S1). This is consistent with the larger contribution from the longer-wavelength ^3^MMLCT emission in the heterometallic complexes, which is less efficient by its nature due to the so-called energy gap law.^63^ We studied complexes **3x**, **4x**, **7**, and **8** at various concentrations, from 10^–7^ to 10^–4^ M to shed some light onto the origins of the long wavelength ^3^MMLCT emission (Figure 6).

**Figure 6:**
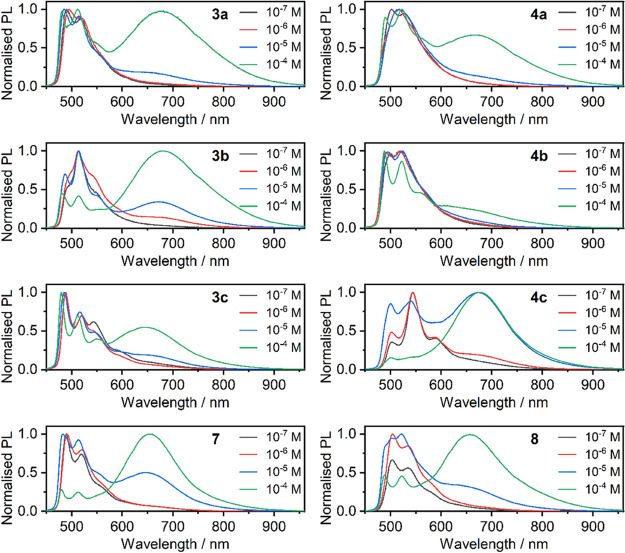
Normalized emission spectra of **3x, 4x, 7**, and **8** recorded in degassed acetonitrile solutions at concentrations indicated in each figure legend, λ_exc_ = 365 nm.

There is a very clear dependence of the ^3^MMLCT PL intensity on concentration, confirming our initial assumption about the *inter*molecular origin of this photoluminescence. While the ^3^MMLCT band behaves in-line with other aggregating or excimer-forming Pt­(II) complexes, the shorter wavelength band displays an altered vibronic structure as concentrations change. The latter is observed for all complexes, except perhaps in **3a** and **3c**, where this effect is negligible. This demonstrates a complex nature of the processes leading to or somewhat correlating with aggregation.

Despite the similarity with the archetypal complex **Pt­(dpyb)-Cl**, there are visible differences between the PL spectra at RT of the former and complexes **3x**, **4x**, **7**, and **8** to a various extent.^49^ Thus, a more sophisticated photophysical picture must be at work.

To gain more insight in this aspect, we studied the photoluminescence of our complexes in 2-methyltetrahydrofuran (2-MeTHF) at 77 K, unravelling a particularly complex excited state behavior (Figure 7). It appears that complexes **7** and **8**, as well as **3a** and **4a** display phosphorescence spectra which are consistent or at least similar to those presented by analogous Pt­(*NCN*)-X complexes.

**Figure 7:**
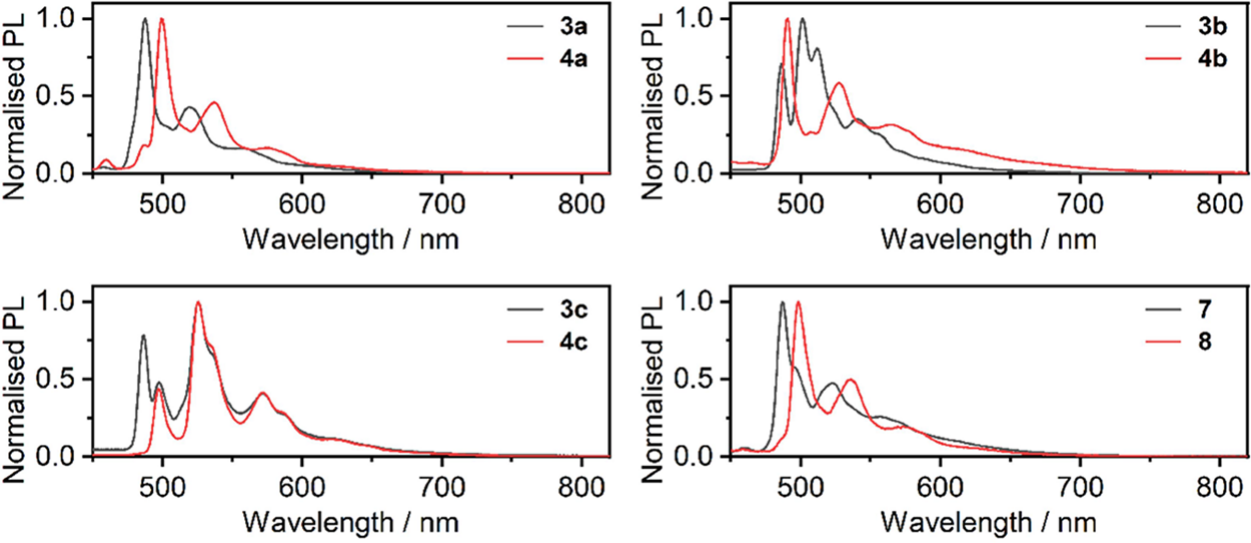
Normalized PL spectra of complexes **3x, 4x, 7**, and **8** recorded in 2MeTHF glass (*c* = 10^–5^ M) at 77 K, λ_exc_ = 365 nm.

These four examples feature a thienyl (**3a** and **4a**) and de facto phenyl (**7** and **8**) connected at the far end of the acetylide ligand. Both of these groups display triplet (T_1_) energy above that of the Pt­(*NCN*)-X chromophore. **3b** as well as **3c**/**4c** display additional spectral features in their photoluminescence to those present in the previously discussed complexes, which can be attributed to phosphorescence originating mainly from the ^3^LC (ligand-centered) state of either of the aromatic units at the far end of the acetylide ligand. Unlike **3b**, **4b** behaves rather similarly to complexes **3a** and **4a**. The behavior of these four complexes deserves further, extended discussion.

We analyzed the PL spectra of **3b**/**4b** as well as **3c**/**4c** recorded in dilute 2-MeTHF solutions at 77 K in the context of the reported phosphorescence spectra of naphthalene and phenanthrene (Figure S13). In the case of naphthalene, we were not able to find the spectrum of the pure hydrocarbon, but rather of its adduct with a mercury complex acting as a Lewis acid. Hence the spectrum is red-shifted to what we assume would be the true phosphorescence spectrum of naphthalene. Nevertheless, the two spectra display similar vibronic patterns to each other and to the **3b**, **3c**, and **4c** at 77 K. This unequivocally demonstrates that the ^3^LC state of the naphthalene or phenanthrene is populated at the expense of the Pt­(*NCN*)-X moiety. We also observe that complexes **4x** display a slightly lower triplet energy of the Pt­(*NCN*)-X moiety than **3x**, which is evidenced through analysis of the PL spectra of pairs **3a**/**4a** and **7**/**8** at 77 K. This assessment explains the divergent behavior of complex **4b** in respect to its counterpart **3b**—the ^3^MXLCT state of the Pt­(*NCN*)-X moiety is in this case below the ^3^LC state of naphthalene. This is also observed in our calculations. In **3b**, **3c**, and **4c** the ^3^LC state is energetically slightly below that of the ^3^MXLCT, acting as a triplet trap. Complexes **3c**/**4c** demonstrate very clearly the contributions of the ^3^LC and ^3^MLCT nature, with the latter evidenced in the bluer part of the PL spectra. We believe that **3b** might be a similar case to **3c** in this respect. The band at λ_PL_ = 486 nm in **3c** resembles that in **3a** and **3b** localized at the same wavelength ± 2 nm, which indicates a common contribution from the Pt­(*NCN*)-X moiety. The other vibronic bands at λ_PL_ = 497, 526, 572 nm in **3c** (and **4c**) can be assigned to the phenanthrene, while bands λ_PL_ = 501, 512, 540 nm in **3b** to naphthalene.

Moreover, certain features of phenanthrene/naphthalene phosphorescence are distinctly observable in the PL of **3c** and **4c** even at RT, whereas these features are less pronounced in **3b**. These results suggest that, at RT, the typical ^3^MLCT emission of the Pt­(NCN)-X group exists in equilibrium with photoluminescence originating from the ^3^LC state. The lack of such behavior in **3a** and **4a** as well as **7** and **8** at RT and 77 K confirms that the energy of the ^3^LC of the aromatic group at the far end of the acetylide ligand must be significantly higher than that of the ^3^MLCT state.

### Time-Resolved Photoluminescence Spectra and Decay Transients

The time-resolved photoluminescent behavior of complexes **3x, 4x, 7**, and **8** in degassed acetonitrile solutions (*c* = 10^–5^ M) was studied to shed light onto the behavior of both the bluer and the more red-shifted components of the PL spectrum (Figure 8). To aid the discussion we divide the molecules into two groups guided by their steady-state behavior: (I) complexes **3a**, **4a**, **4b**, **7**, and **8**, and (II) complexes **3b**, **3c**, and **4c**. These two groups differ in the luminescent behavior of the ^3^MXLCT emission, but the behavior of the ^3^MMLCT emission is common to all of them. In this respect only complexes **3c** and **4b** do not display detectable ^3^MMLCT emission.

**Figure 8:**
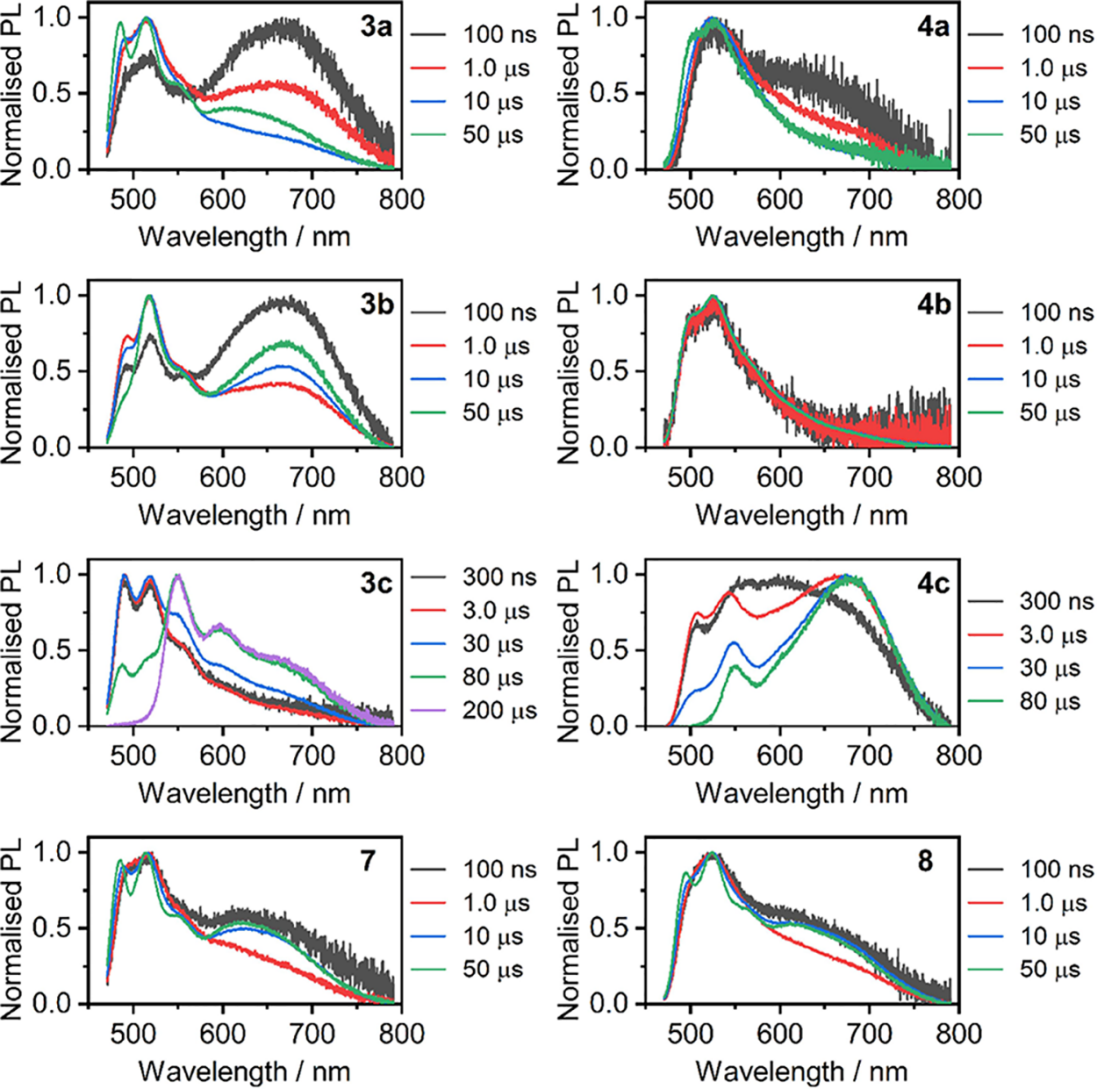
Transient photoluminescence spectra recorded in degassed acetonitrile solutions (*c* = 10^–5^ M) at RT (λ_exc_ = 355 nm). Delay times are indicated in each figure legend.

In group (I) complexes there is very little change in the ^3^MXLCT or monomeric emission over time. On the contrary, in group (II) there can effectively be observed two luminescent bands originating from the isolated Pt­(NCN)-X units: one that resembles the normal ^3^MXLCT emission and the other one, that can be attributed to the ^3^LC emission (or a mixed ^3^MXLCT + ^3^LC PL) associated with the terminal aromatic unit. These two emissions may originate either from the complex kinetics between the emissive ^3^MXLCT and ^3^LC states or from different conformations of the same molecules.

Generally speaking, the PL attributed to the ^3^MMLCT state displays a behavior in which the emission at first decays slightly faster than the ^3^MXLCT PL, evidenced by its reducing contribution over time, to then also display a rise in intensity at longer decay times. This indicates the ^3^MMLCT PL originates from two different sources, each of them giving rise to two different decay regimes. This behavior is akin to what some of the current authors have observed earlier in Pt­(II) complexes.^49,64^ For example, somewhat related ‘sandwich’ complexes of analogous *NCN* cyclometallating ligands display short Pt···Pt contacts leading to ^3^MMLCT excited states.^56^ In this case we find that the said states form in the ground state or immediately following excitation, but some molecules may form other type of an excited state in which no short Pt···Pt contacts exist. As a result, the ^3^MLCT and ^3^MMLCT emissions decay at different rates. Generally speaking, for true excimers there is a considerable build-in of PL intensity in the 0.1–1 μs time scale.^49^ At the same time the *monomer* and *excimer* components decay with the same lifetimes due to them being associated kinetically. Neither of these is observed in our results, suggesting that the observed ^3^MMLCT PL must originate from (intermolecular) aggregates. This is consistent with the ^3^MMLCT PL being favored at higher concentrations. These conclusions apply to the fast-decaying ^3^MMLCT PL. As to the longer-lived red/NIR PL we speculate that it might be of excimer origin. Unfortunately, the longer-lived PL is generally too weak to determine whether the two luminescent components (*monomer* and supposed *excimer*) indeed decay at similar rates.

### Photoluminescence in Powder

All the presented complexes form dark-colored powders and display a deep red or NIR photoluminescence. Hence, we thought that this property might complement the already very interesting photophysics of these luminophores. Our results are summarized in Table 2, while pertinent photoluminescence spectra recorded at RT and 77 K are shown in Figure 9.

**Table 2:** Photoluminescence Characteristics of Complexes **3a**/**4a**, **3b**/**4b**, **3c**/**4c**, and **7**/**8** in Powder at Room Temperature and 77 K

compound	λ_PL_@ RT/nm	λ_PL_@ 77K/nm	Φ_PL_	τ (μs)
**3a**	688	749	0.03	0.35
**3b**	708	697, 800sh	0.09	0.48
**3c**	693	642sh, 732	0.07	0.33
**7**	721	694sh, 778	0.06	0.38
**4a**	686	699, 768sh	0.01	0.41
**4b**	494^a^, 668	637, 719, 763sh	0.04	0.49
**4c**	689, 820sh	687	0.05	0.31
**8**	707	685, 780	0.03	0.44

a Monomer emission.

**Figure 9:**
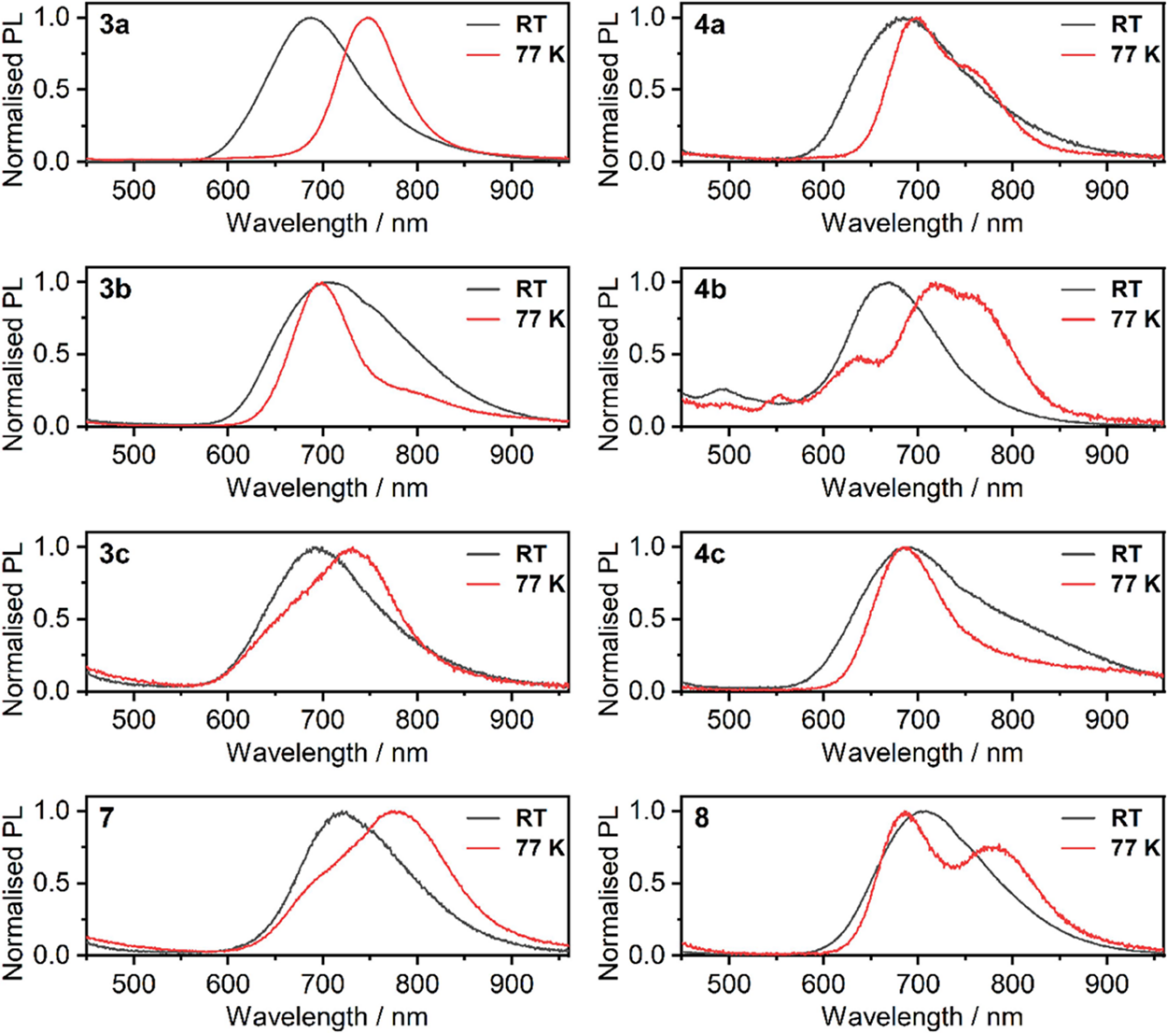
Normalized emission spectra of complexes **3x, 4x, 7**, and **8** in powder RT and 77 K. λ_exc_ = 365 nm.

All complexes display broadband photoluminescence at λ_PL_ ∼ 650–750 nm which resembles the typical ^3^MMLCT emission of analogous Pt­(*NCN*)-Cl complexes in neat films.^65^ Such excited states in solids are usually formed by ground state short Pt···Pt contacts, hence can be attributed to aggregation of the Pt­(*NCN*)-X units (be it an *intra*- or *inter*molecular interaction).^56^ Φ_PL_ values are relatively modest, < 0.1, with the corresponding photoluminescence lifetimes, τ, in the order of 0.3–0.5 μs. These figures are generally in agreement with the ^3^MMLCT emission of the parent Pt­(*NCN*)-Cl complexes.

The ^3^MMLCT emission of all complexes at RT agrees with that of the parent Pt­(*NCN*)-Cl complexes, but for **3b**, **7**, and **8** the λ_PL_ is visibly shifted more into the NIR in respect to the other complexes. Furthermore, we observe a long wavelength shoulder in the PL of **4c** at ∼750–900 nm. These observations suggest that, apart from the conventional dimeric Pt···Pt interactions, other interactions must also occur. Hence, such emissions suggest the presence of excited states spanning multiple Pt­(*NCN*)-X units. Further investigation into this behavior reveals that at 77 K some of the samples display multiple emissive bands. Considering that generally ^3^MMLCT emission spectra of platinum­(II) complexes resemble bell curves the occurrence of two or more maxima or additional emission shoulders at 77 K is strongly indicative of the presence of dimers as well as larger aggregates. It should be considered that analogous Pt­(*NCN*)-X complexes display similar behaviors.^49,55^

Pt···Pt interactions are a complex matter with the dimers and excimers often existing with one another at certain conditions. However, the dark color of the samples and a general susceptibility of flat platinum complexes to form short Pt···Pt contacts in the ground state in films unequivocally indicates dimer-like interactions in our case. Here, by aggregates we understand interacting Pt­(*NCN*)-X units, whether in an *intra*- or *inter*molecular fashion. Dimeric emissions may originate from either *intra*- or *inter*molecular interactions of the Pt­(II) centers, while higher order aggregates require a combination of *inter*- and *intra*molecular contacts or a sandwich-like interaction. Having this said, we believe that the 2-fold larger quantity of Pt­(*NCN*)-X units in complexes **7** and **8** facilitates *inter*molecular contacts, leading to more likely formation of higher order aggregates. To sum up, the behavior of our complexes in solid state does not generally correlate with their structure (except perhaps for complexes **7** and **8**). Their particular behavior in solid state is generally dominated by specific intermolecular interactions and molecular packing.

### Light-Emitting Electrochemical Cells (LEECs) and Electrochemistry of **4c**

Considering factors such as solubility, material availability, and interesting photophysics we have selected compound **4c** to serve as an example to demonstrate the potential application of our ionic heteronuclear complexes in proof-of-concept light-emitting electrochemical cells (LEECs).

Before attempting to produce LEECs we have studied the electrochemical response of the of **4c** in 0.1 M tetrabutylammonium tetrafluoroborate (TBABF_4_)/CH_2_Cl_2_. Somewhat expectedly for monoplatinum­(II) complexes of this type, we observed irreversible oxidation and reduction waves (Figure S23). In both cases the irreversible process leads to adsorption/deposition of new species on the surface of the working electrode, leading to different electrochemical responses in first and consecutive cycles.

The structure of the cell follows the recently reported highly efficient Ir­(III) complex-based LEECs:^66^ ITO | PEDOT:PSS (AI4083) (30 nm) | PVK:OXD7:THABF_4_ (56:38:6) co 4% **4c** (100 nm) | Al (100 nm). The blend of PVK {poly­(9-vinylcarbazole)} and OXD7 {1,3-bis­[2-(4-*tert*-butylphenyl)-1,3,4-oxadiazo-5-yl]­benzene} plays a role of a host matrix which is doped with THABF_4_ (tetrahexylammonium tetrafluoroborate) electrolyte.

The device was driven at a constant voltage bias as previously described.^67^ The LEEC demonstrated a turn-on at ∼11–12 V and was driven at 12.5 and 13 V, respectively. The electroluminescence spectrum of the LEEC (Figure 10a) is representative of the photoluminescence of **4c** in dilute solutions, which can be rationalized with the low emitter concentration in the emissive layer. PL spectrum of the EML is presented in Figure S24. Given our earlier discussion of **4c** photophysics, we note that most of the EL originates from the triplet state associated with the phenanthrene unit (λ_EL_ = 556 nm).

**Figure 10:**
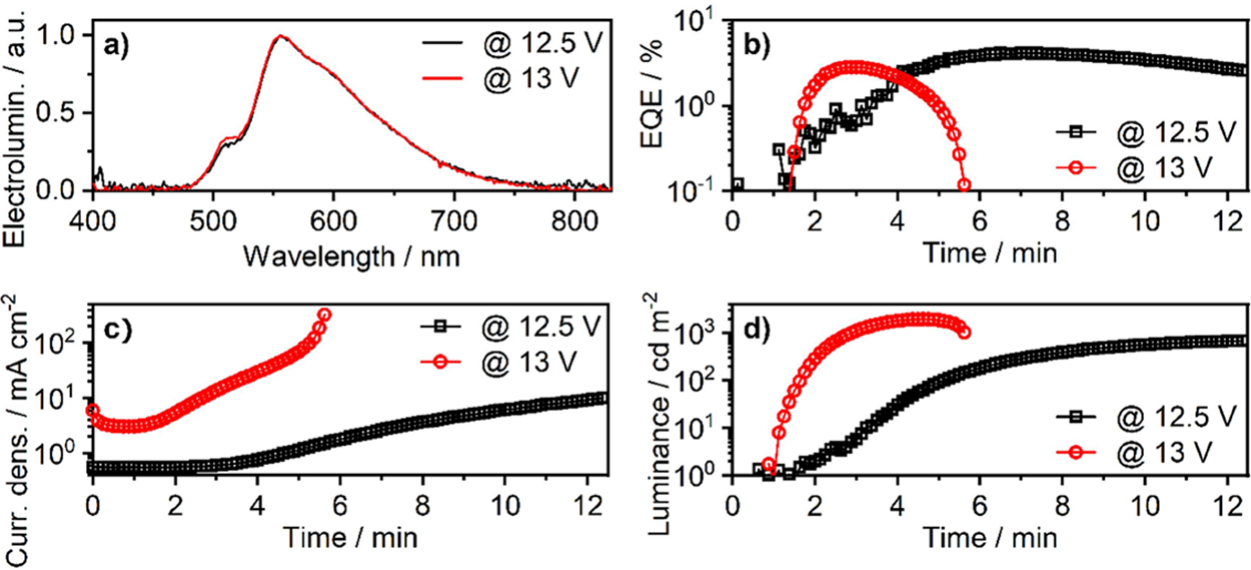
Characteristics of LEECs featuring **4c** as the emitter recorded at two different driving voltages: (a) EL spectra; (b) external quantum efficiency (EQE); (c) current density; (d) luminance.

The LEEC driven at 12.5 V displays a maximum luminance of 690 cd m^–2^ at t = 12.5 min and maximum EQE of 4.1%, at t = 7.1 min. Driving the LEEC at 13 V yields a higher maximum luminance of 1970 cd m^–2^ at t = 4.6 min, but a lower maximum EQE of 2.8%, at *t* = 3.1 min (Figure 10b–d). These characteristics are comparable with the best results reported for the most popular ionic iridium­(III) complexes.^68^ LEEC emitters are generally dominated by iridium­(III) luminophores, likely due to their high photoluminescence efficiency and relatively simple introduction of the ionic structure. A very limited number of other, mainly copper­(I)^69^ and silver­(I),^70^ complexes have been used in LEECs and a small number of metal-free thermally activated delayed fluorescence (TADF) emitters have also been reported as LEEC emitting dopants, with various results.^14,71,72^

Nevertheless, platinum­(II) complexes have not been widely studied as LEEC luminescent dopants. To the best of our knowledge only one such work exists, but EQE and luminance have not been reported.^73^ There have been however no reports of LEECs that would display appreciable luminance using a platinum­(II) or a mixed silver­(I)/platinum­(II) complex emitter. Given that efficient and luminous LEECs are scarce and there exist very few efficient (EQE > 2–4%) and bright (luminance >1000 cd m^–2^) examples, these results are competitive with the best reported so far and definitely unique for platinum­(II) complexes.

## Conclusions

This study reports the synthesis and characterization of eight novel Pt–Ag heteronuclear ionic complexes derived from Pt­(NCN)-C≡CR precursors. These complexes exhibit red-to-NIR photoluminescence (650–750 nm) due to an interplay of ^3^MLCT, ^3^LC, and ^3^MMLCT states, with aggregation-induced emissions in the solid state driven by short Pt···Pt contacts. The nature of the aryl substituent on the acetylide ligand significantly influences the excited-state behavior and photophysical properties.

The practical potential of these complexes was demonstrated using **4c** as an emitter in proof-of-concept LEECs, achieving a maximum EQE of 4.1% and luminance of 1970 cd m^–2^. These results rank among the best reported for Pt-based LEECs, emphasizing the role of ligand design in tuning optical and electroluminescent properties.

This work underscores the versatility of Pt–Ag heteronuclear complexes in light-emitting applications, offering a platform for designing efficient, tunable emitters. By bridging fundamental photophysics and device development, these findings position Pt-based systems as promising alternatives to conventional iridium-based emitters in sustainable light-emitting technologies.

## Supplementary Material



## Notes

The data underlying this study are openly available in Zenodo at 10.5281/zenodo.15038518. The Supporting Information is available free of charge at https://pubs.acs.org/doi/10.1021/acsami.4c22938.

ESI–MS^+^ spectra for all compounds, additional photophysical data (titration experiments, quantum yields and lifetimes), and DFT calculations (optimized geometries and relevant orbital iso-surfaces) (PDF)
