# Supplementary material for: Heteronuclear Complexes Containing Pt(II) and Ag(I) Centers: Application to Efficient Light-Emitting Electrochemical Cells
Source: ACS Appl Mater Interfaces. 2025 May 19;17(21):31064–77. doi: 10.1021/acsami.4c22938 (PMC12163895; doi:10.1021/acsami.4c22938)
Supplement: Supplementary file 1 [file am4c22938_si_001.pdf]

Supporting Information

Heteronuclear Complexes Containing Pt(II) and Ag(I) Centers: Application to Efficient Light-Emitting Electrochemical Cells

Ariadna Lázaro,[a,b] Margarita Crespo,[a,c] Piotr Pander,[d,e*] Fernando B. Dias,[f*] and Laura Rodríguez[a,b*]

[a] *Departament de Química Inorgànica i Orgànica, Secció de Química Inorgànica, Universitat de Barcelona, Martí i Franquès 1-11, E-08028 Barcelona, Spain. e-mail: laurarodriguezr@ub.edu*

[b] *Institut de Nanociència i Nanotecnologia (IN2UB). Universitat de Barcelona, 08028 Barcelona, Spain*

[c] *Institut de Biomedicina de la Universitat de Barcelona (IBUB), 08028-Barcelona, Spain*

[d] *Faculty of Chemistry, Silesian University of Technology, M. Strzody 9, 44-100 Gliwice, Poland. e-mail: Piotr.Pander@polsl.pl*

[e] *Centre for Organic and Nanohybrid Electronics, Silesian University of Technology, Konarskiego 22B, 44-100 Gliwice, Poland*

[f] *Department of Physics, Durham University, South Road, Durham, UK, DH1 3LE. e-mail: f.m.b.dias@durham.ac.uk*

1 Mass spectra

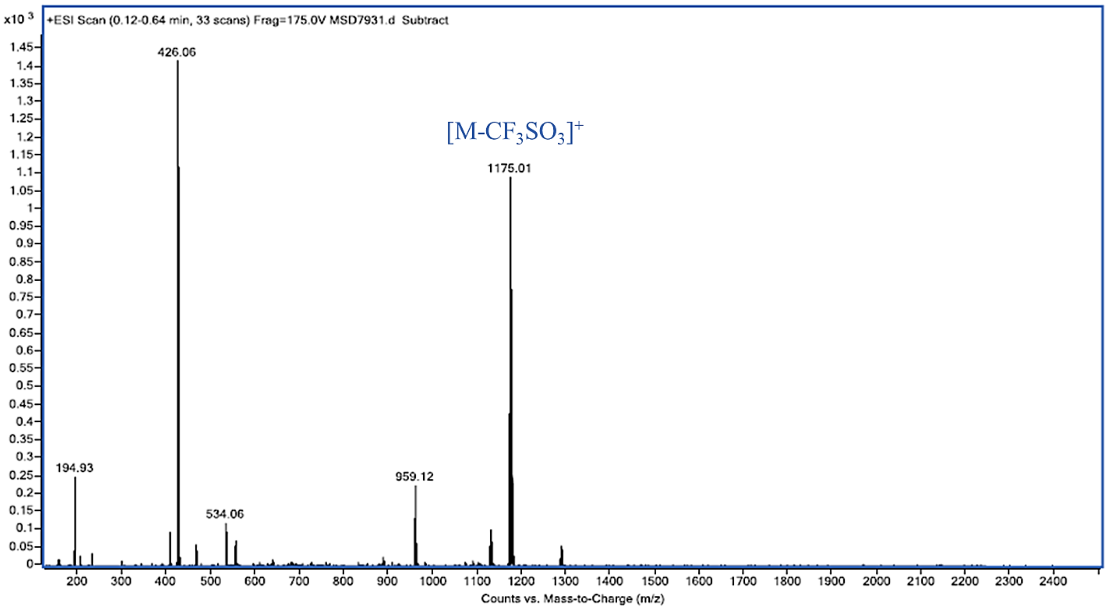

Figure S1. ESI-MS(+) spectrum of **3a**.

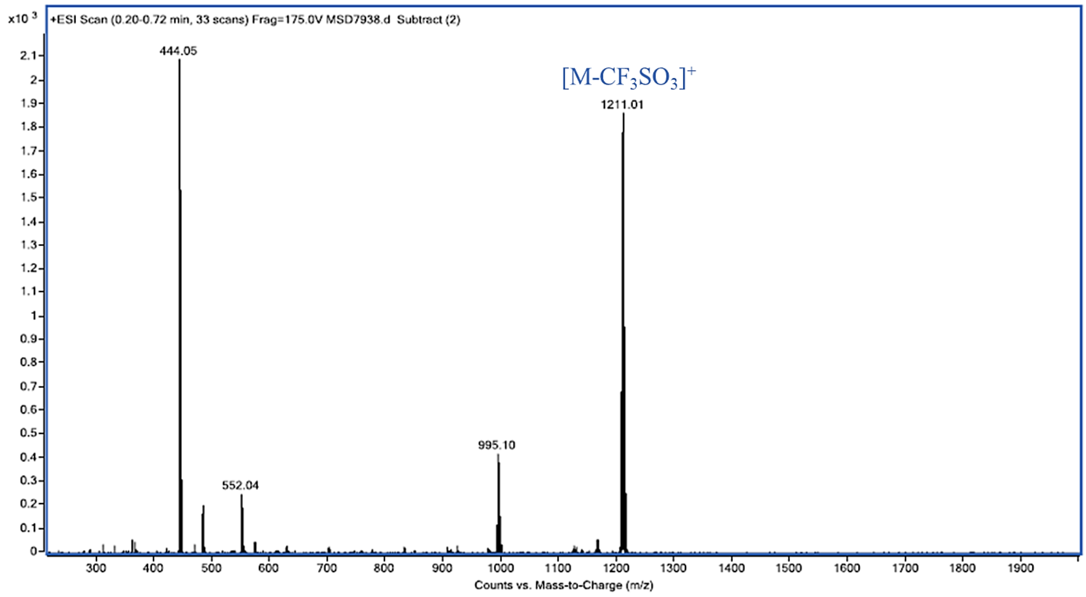

Figure S2. ESI-MS(+) spectrum of **4a**.

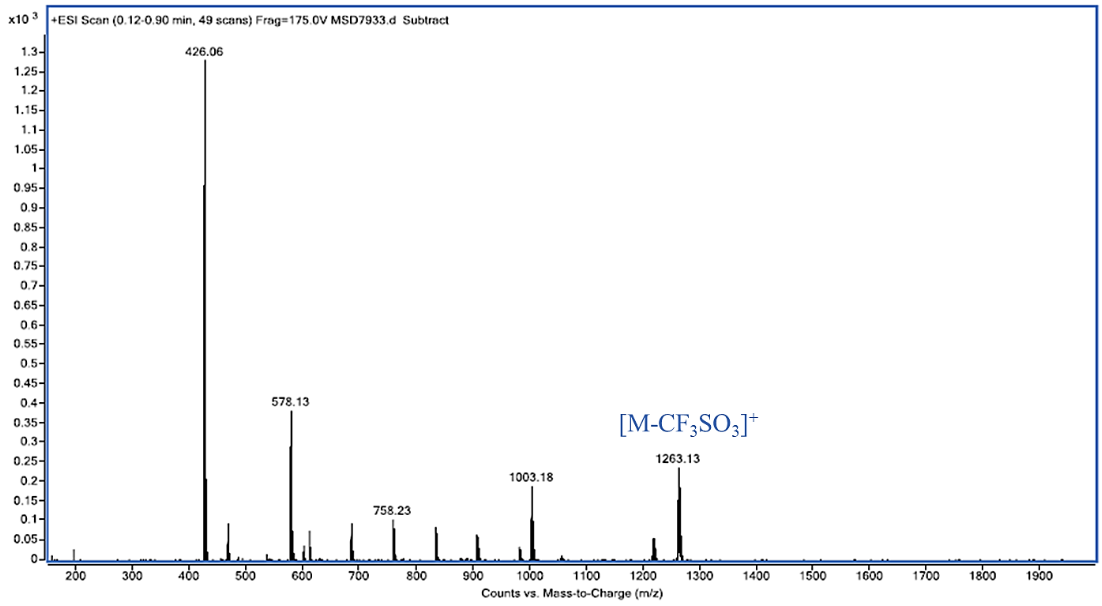

Figure S3. ESI-MS(+) spectrum of **3b**.

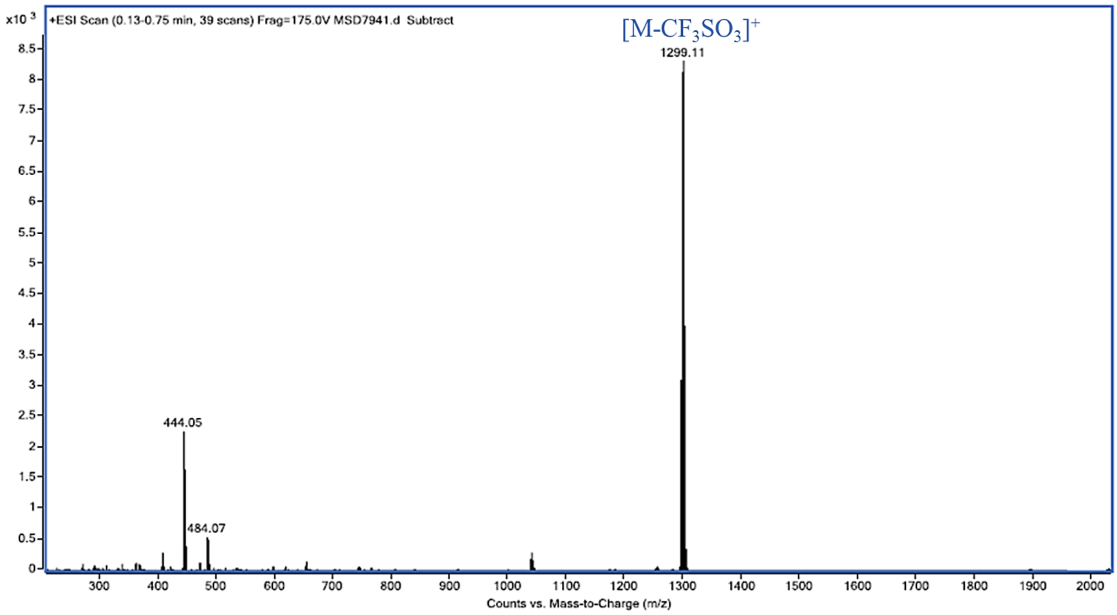

Figure S4. ESI-MS(+) spectrum of **4b**.

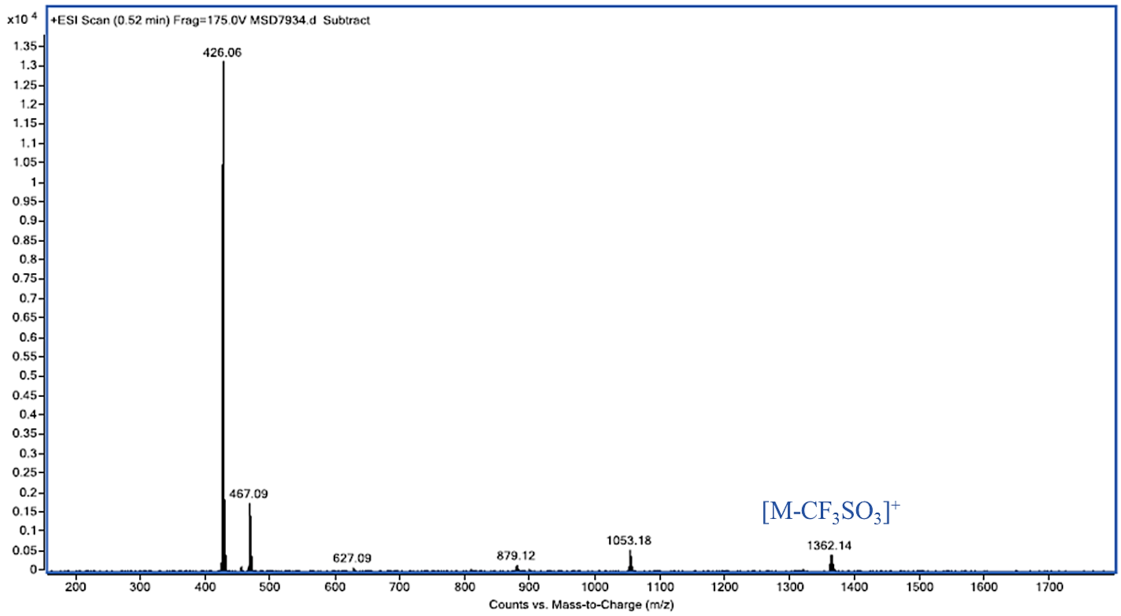

Figure S5. ESI-MS(+) spectrum of **3c**.

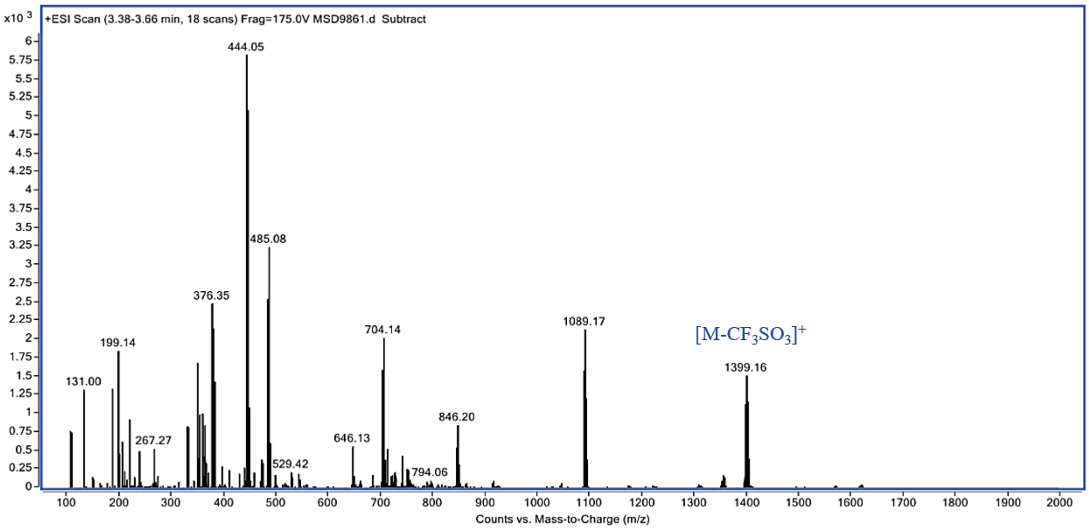

Figure S6. ESI-MS(+) spectrum of **4c**.

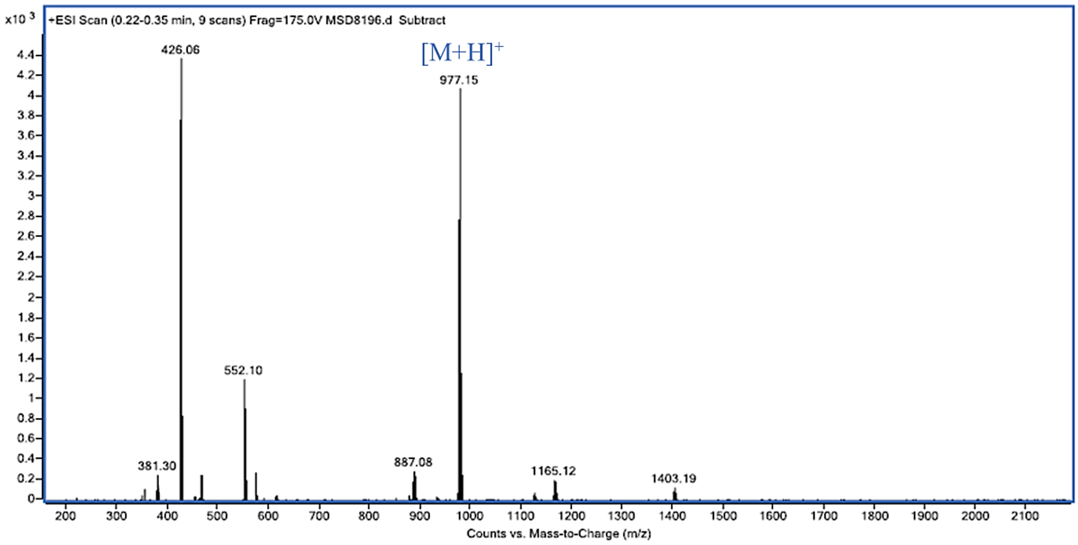

Figure S7. ESI-MS(+) spectrum of **5**.

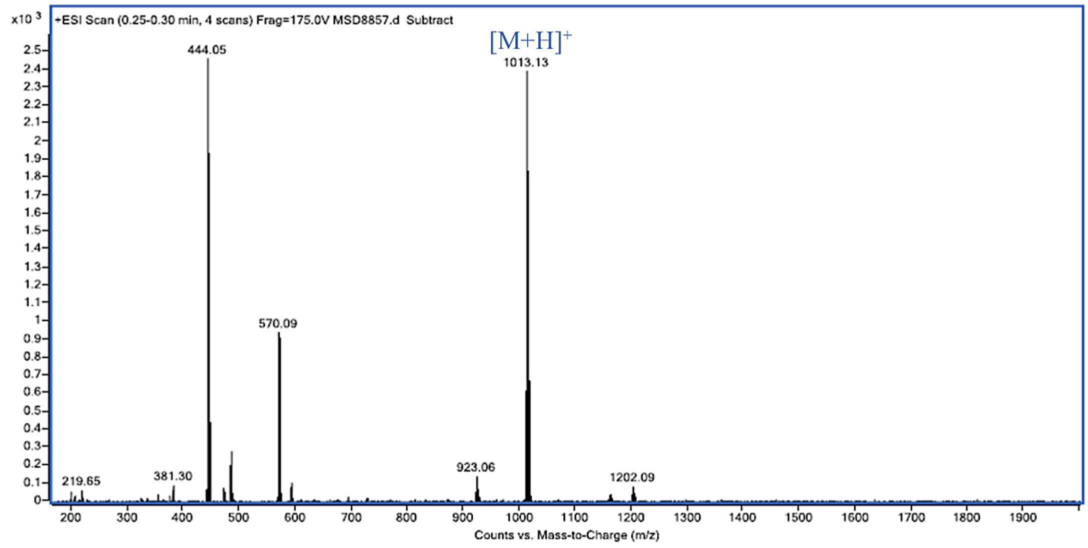

Figure S8. ESI-MS(+) spectrum of **6**.

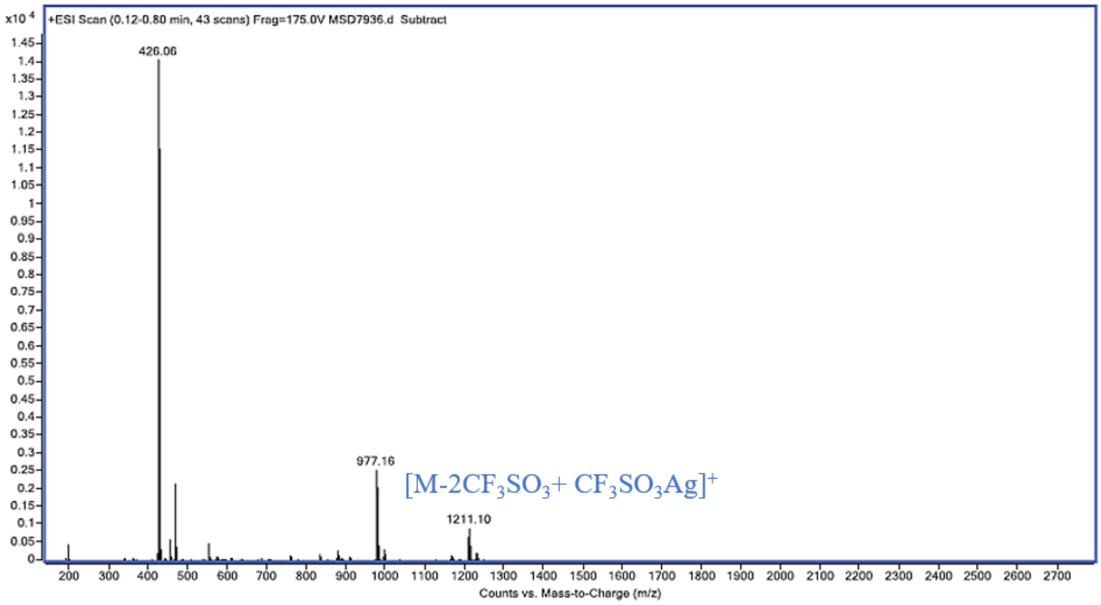

Figure S9. ESI-MS(+) spectrum of **7**.

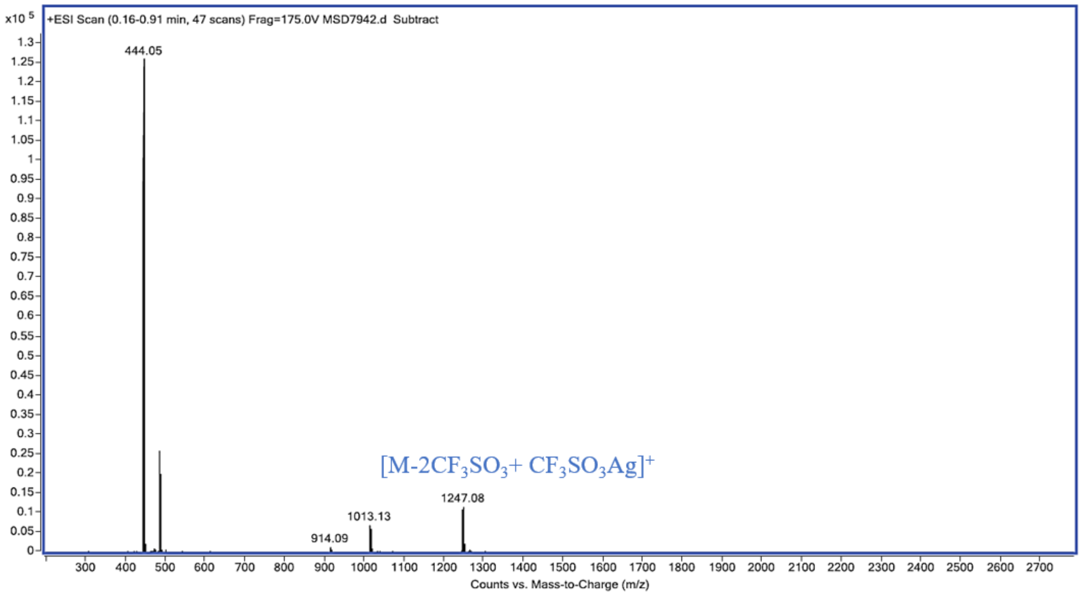

Figure S10. ESI-MS(+) spectrum of **8**.

2 Photophysics

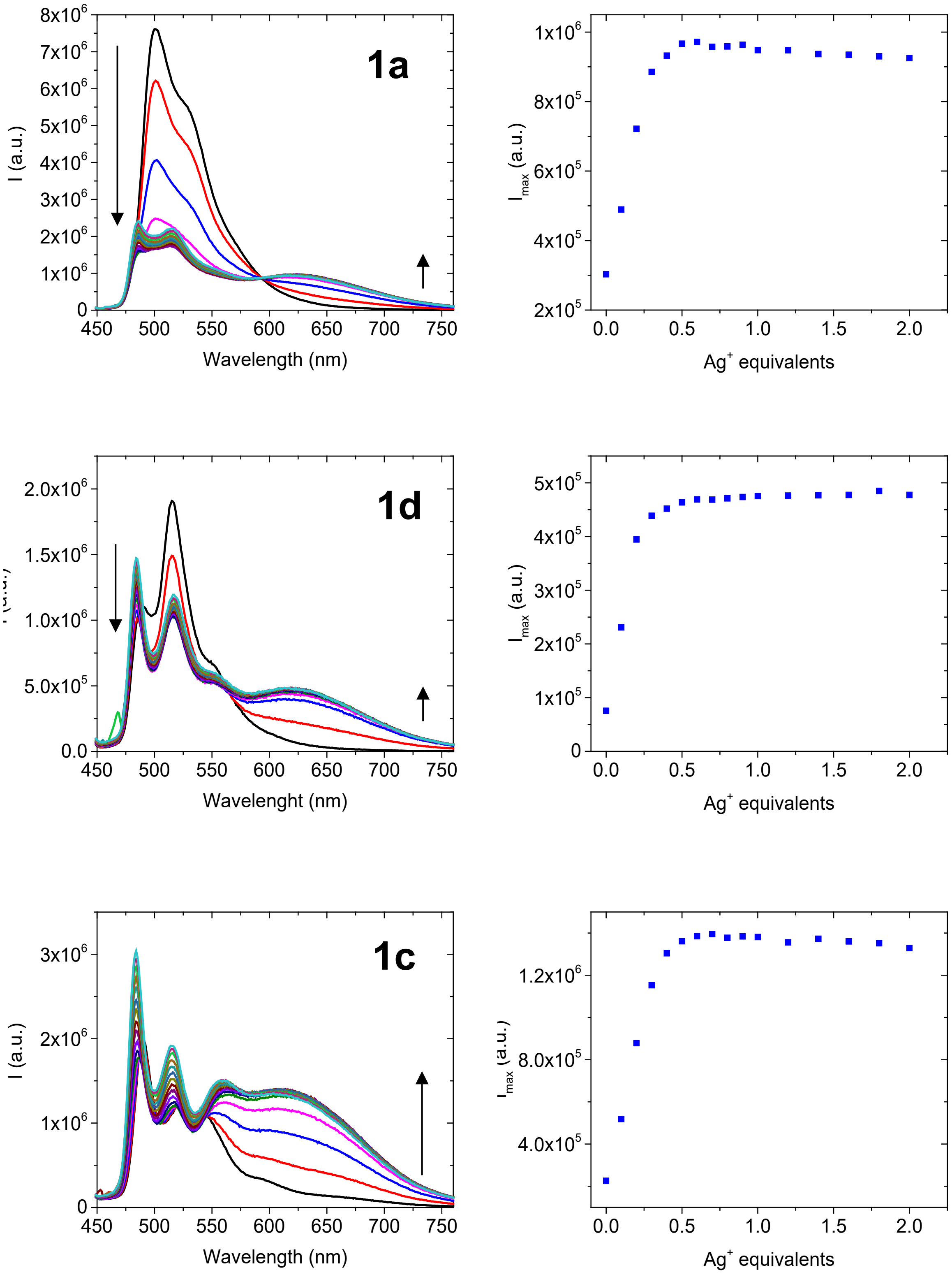

Figure S11. Emission titration data of compounds **1x** with increasing amounts of silver triflate (λ_{exc} = 390 nm) (left) and plot of the new emission maxima (λ_{em} = 625 nm) against number of equivalents of silver(I) salt (right).

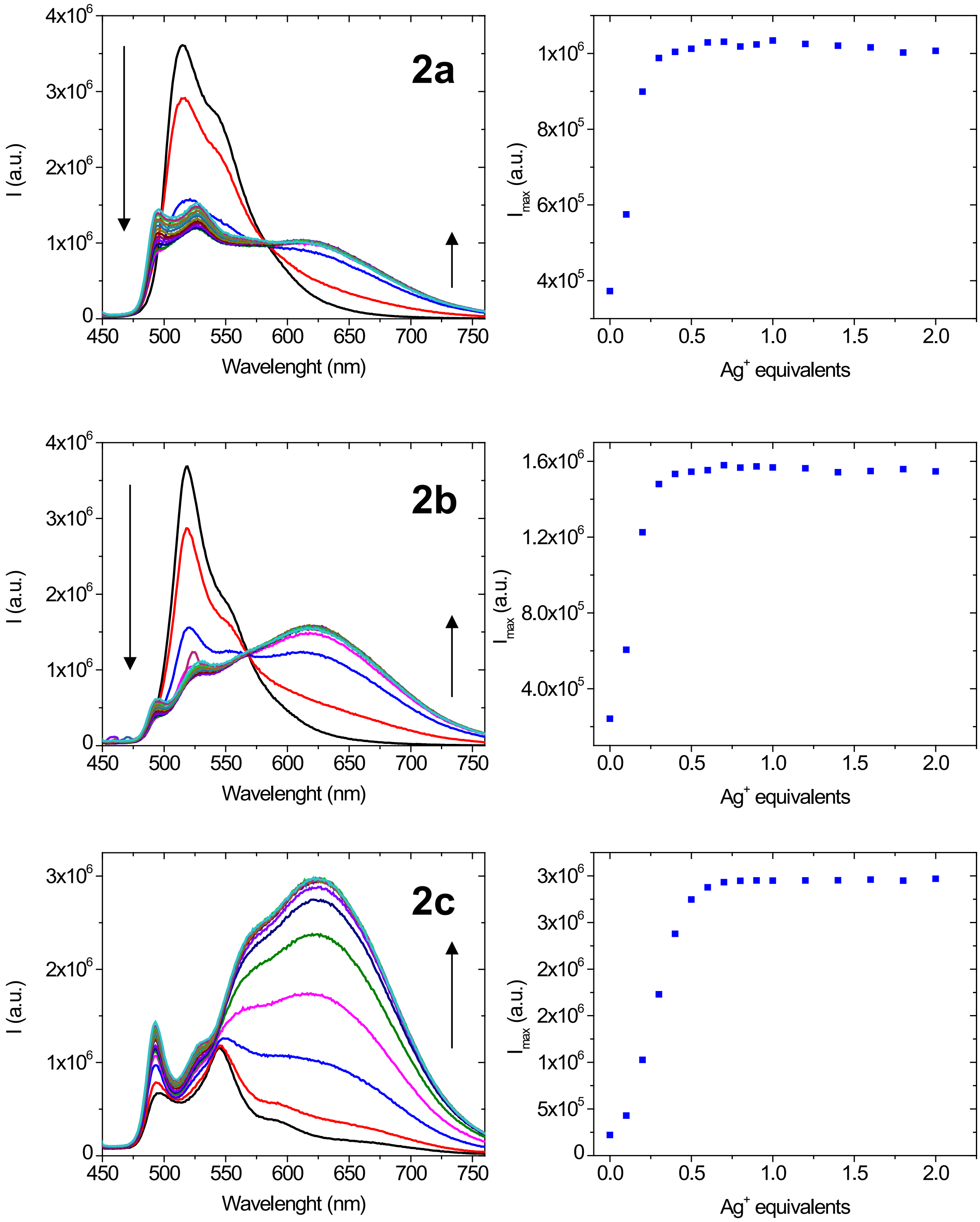

Figure S12. Emission titration data of compounds **2x** with increasing amounts of silver triflate (λ_{exc} = 390 nm) (left) and plot of the new emission maxima (λ_{em} = 625 nm) against number of equivalents of silver(I) salt (right).

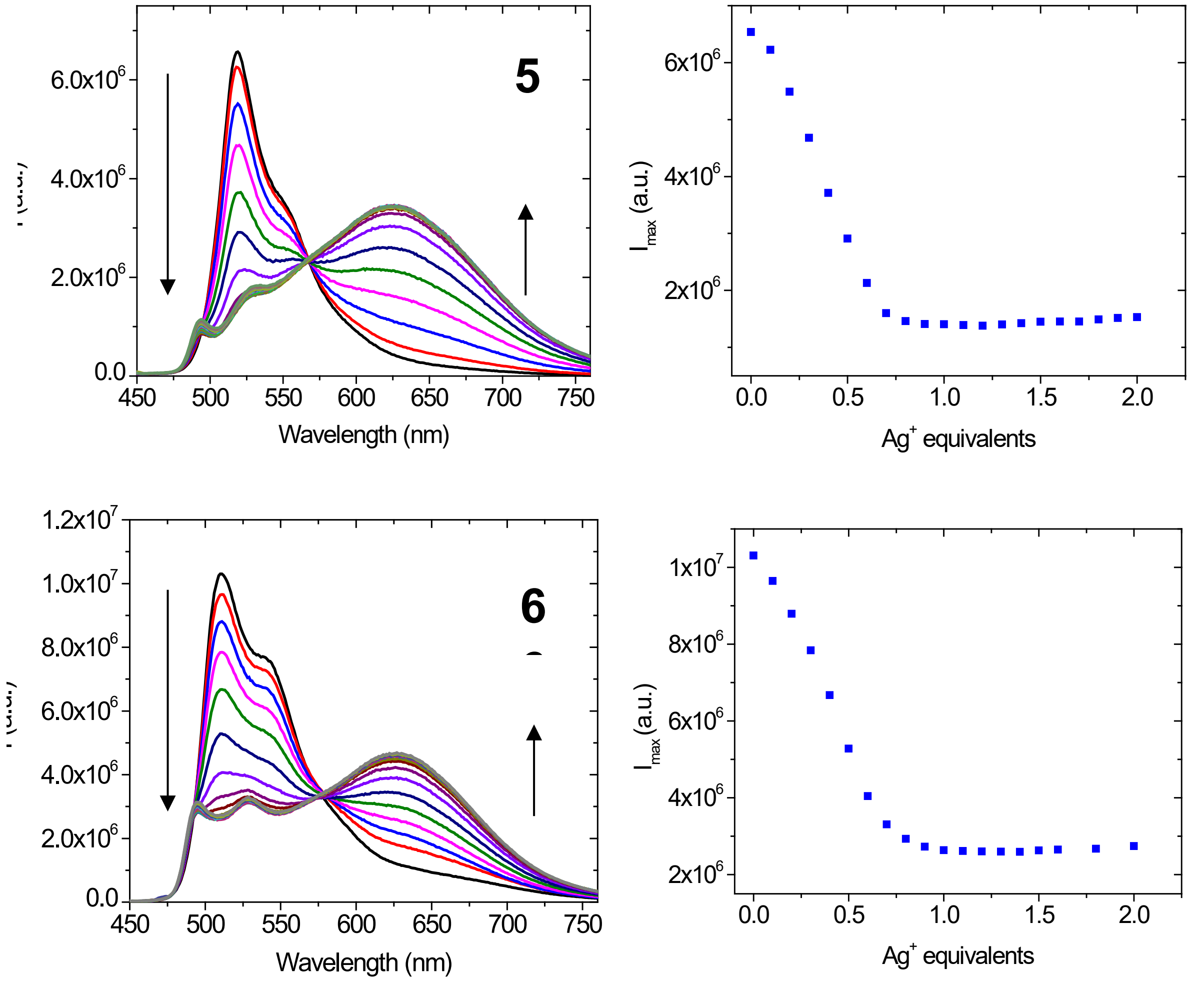

Figure S13. Emission titration data of compounds **5** and **6** with increasing amounts of silver triflate (λ_{exc} = 390 nm) (left) and plot of the decrease in emission maxima (λ_{em} = 525 nm) against number of equivalents of silver(I) salt (right).

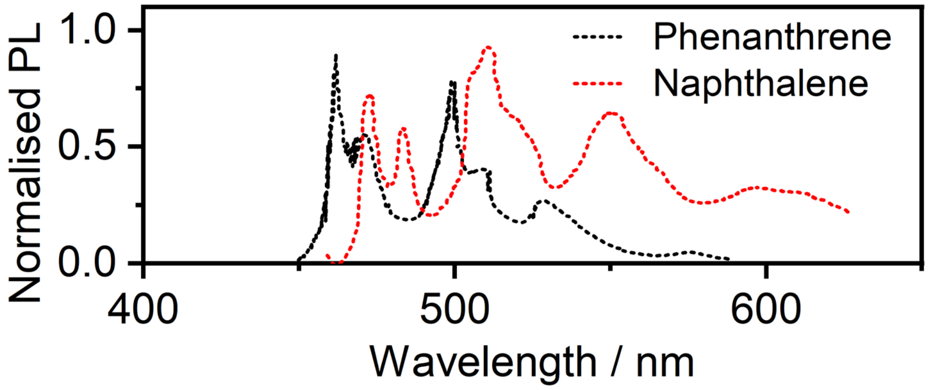

Figure S14. Phosphorescence spectra of phenanthrene[1] and naphthalene complex[2] reproduced from the original articles.

Table S1. Photoluminescence quantum yield and radiative lifetimes recorded in dilute (c = 10^{-5} M) degassed acetonitrile solutions at 298 K.

**Compound**	Φ_{PL}	**τ (μs)** **λ_{col} = 500 nm**	**τ (μs)** **λ_{col} = 680 nm**
**3a**	0.38	5.42	5.99
**3b**	0.36	4.81	7.12
**3c**	0.32	6.76	7.35
**7**	0.36	4.97	8.70
**4a**	0.27	5.97	6.66
**4b**	0.21	6.73	7.33
**4c**	0.29	3.75	7.55
**8**	0.27	3.85	5.25

3 Calculations

Table S2. Characteristics of the three lowest singlet and triplet excitations at the T_1 geometry of **3a**.

**State**	**Orbital pairing**	**Character**	**Energy, eV**
**S_1**	HOMO→LUMO (96%)	MXLCT $d_{Pt(1,2)} + d_{Ag} + \pi_{C\equiv CAr} \rightarrow \pi_{dpyb}^*$	2.471
**S_2**	HOMO-1→LUMO (94%)	MXLCT $d_{Pt(1,2)} + d_{Ag} + \pi_{C\equiv CAr} + \pi_{dpyb} \rightarrow \pi_{dpyb}^*$	2.745
**S_3**	HOMO-2→LUMO (92%)	MXLCT $d_{Pt(1,2)} + d_{Ag} + \pi_{C\equiv CAr} + \pi_{dpyb} \rightarrow \pi_{dpyb}^*$	2.860
**T_1**	HOMO→LUMO (93%)	MXLCT $d_{Pt(1,2)} + d_{Ag} + \pi_{C\equiv CAr} \rightarrow \pi_{dpyb}^*$	2.423
**T_2**	HOMO-1→LUMO (55%) HOMO-2→LUMO (26%)	MXLCT $d_{Pt(1,2)} + d_{Ag} + \pi_{C\equiv CAr} + \pi_{dpyb} \rightarrow \pi_{dpyb}^*$	2.510
**T_3**	HOMO-3→LUMO (39%) HOMO-2→LUMO (16%)	MXLCT $d_{Pt(1,2)} + d_{Ag} + \pi_{C\equiv CAr} + \pi_{dpyb} \rightarrow \pi_{dpyb}^*$	2.660

Table S3. Characteristics of the three lowest singlet and triplet excitations at the T_1 geometry of **3b**.

**State**	**Orbital pairing**	**Character**	**Energy, eV**
**S_1**	HOMO→LUMO (95%)	MXLCT<br>$d_{Pt(1,2)} + d_{Ag} + \pi_{C\equiv CAr} \rightarrow \pi_{dpyb}*$	2.498
**S_2**	HOMO-1→LUMO (83%)<br>HOMO-2→LUMO (13%)	MXLCT<br>$d_{Pt(1,2)} + d_{Ag} + \pi_{C\equiv CAr} + \pi_{dpyb} \rightarrow \pi_{dpyb}*$	2.724
**S_3**	HOMO-2→LUMO (83%)<br>HOMO-1→LUMO (11%)	MXLCT + ILCT<br>$d_{Pt(1,2)} + d_{Ag} + \pi_{C\equiv CAr(1)} + \pi_{dpyb} \rightarrow \pi_{dpyb}* + \pi_{C\equiv CAr(2)}*$	2.854
**T_1**	HOMO→LUMO (74%)	MXLCT<br>$d_{Pt(1,2)} + d_{Ag} + \pi_{C\equiv CAr} \rightarrow \pi_{dpyb}*$	2.440
**T_2**	HOMO→LUMO+5 (72%)<br>HOMO-1→LUMO+5 (10%)	MXLCT + LC + ILCT<br>$d_{Pt(1,2)} + d_{Ag} + \pi_{C\equiv CAr(1,2)} + \pi_{dpyb} \rightarrow \pi_{C\equiv CAr(2)}*$	2.497
**T_3**	HOMO-2→LUMO (40%)<br>HOMO-1→LUMO (25%)<br>HOMO→LUMO (14%)	MXLCT<br>$d_{Pt(1,2)} + d_{Ag} + \pi_{C\equiv CAr} + \pi_{dpyb} \rightarrow \pi_{dpyb}*$	2.518

Table S4. Characteristics of the three lowest singlet and triplet excitations at the T_1 geometry of **3c**.

State	Orbital pairing	Character	Energy, eV
**S_1**	HOMO→LUMO (96%)	MXLCT<br>$d_{Pt(1)} + d_{Ag} + \pi_{C\equiv CAr(1)} \rightarrow \pi_{dpyb}*$	2.441
**S_2**	HOMO-1→LUMO (90%)	MXLCT<br>$d_{Pt(2)} + d_{Ag} + \pi_{C\equiv CAr(2)} \rightarrow \pi_{dpyb}*$	2.675
**S_3**	HOMO→LUMO+1 (75%)<br>HOMO-2→LUMO (18%)	MXLCT<br>$d_{Pt(1,2)} + d_{Ag} + \pi_{C\equiv CAr(1)} + \pi_{dpyb} \rightarrow \pi_{dpyb}*$	2.916
**T_1**	HOMO→LUMO (46%)<br>HOMO→LUMO+5 (21%)<br>HOMO→LUMO+4 (13%)	MXLCT + LC + ILCT<br>$d_{Pt(1)} + d_{Ag} + \pi_{C\equiv CAr(1)} \rightarrow \pi_{dpyb}* + \pi_{C\equiv CAr(1,2)}*$	2.308
**T_2**	HOMO-1→LUMO (42%)<br>HOMO→LUMO+5 (27%)	MXLCT + LC<br>$d_{Pt(1,2)} + d_{Ag} + \pi_{C\equiv CAr(1,2)} + \pi_{dpyb} \rightarrow \pi_{dpyb}* + \pi_{C\equiv CAr(1,2)}*$	2.369
**T_3**	HOMO→LUMO (45%)<br>HOMO-1→LUMO (19%)<br>HOMO→LUMO+5 (17%)	MXLCT + LC<br>$d_{Pt(1,2)} + d_{Ag} + \pi_{C\equiv CAr(1,2)} + \pi_{dpyb(2)} \rightarrow \pi_{dpyb(1,2)}* + \pi_{C\equiv CAr(1,2)}*$	2.481

Table S5. Characteristics of the three lowest singlet and triplet excitations at the T_1 geometry of **7'**.

State	Orbital pairing	Character	Energy, eV
S_1	HOMO→LUMO (98%)	MXLCT $d_{Pt(1,2)} + d_{Ag} + \pi_{C\equiv CAr(1,2)} + \pi_{dpyb(1,2)} \rightarrow \pi_{dpyb}*$	2.609
S_2	HOMO-1→LUMO (88%)	MXLCT $d_{Pt(1,2)} + d_{Ag} + \pi_{C\equiv CAr(2)} + \pi_{dpyb(1)} \rightarrow \pi_{dpyb}*$	2.837
S_3	HOMO-2→LUMO (87%)	MXLCT $d_{Pt(1,2)} + d_{Ag} + \pi_{C\equiv CAr(1)} + \pi_{dpyb(2)} \rightarrow \pi_{dpyb}*$	2.952
T_1	HOMO→LUMO (79%)	MXLCT $d_{Pt(1,2)} + d_{Ag} + \pi_{C\equiv CAr(1,2)} + \pi_{dpyb(1,2)} \rightarrow \pi_{dpyb}*$	2.407
T_2	HOMO-1→LUMO (31%) HOMO-2→LUMO+2 (29%) HOMO→LUMO+2 (15%)	MXLCT $d_{Pt(1,2)} + d_{Ag} + \pi_{C\equiv CAr(1,2)} + \pi_{dpyb(1,2)} \rightarrow \pi_{dpyb}*$	2.609
T_3	HOMO-1→LUMO (35%) HOMO-2→LUMO (34%) HOMO→LUMO (11%)	MXLCT $d_{Pt(1,2)} + d_{Ag} + \pi_{C\equiv CAr(1,2)} + \pi_{dpyb(1,2)} \rightarrow \pi_{dpyb}*$	2.693

Table S6. Characteristics of the three lowest singlet and triplet excitations at the T_1 geometry of **4a**.

State	Orbital pairing	Character	Energy, eV
**S_1**	HOMO→LUMO (96%)	MXLCT $d_{Pt(1,2)} + d_{Ag} + \pi_{C\equiv CAr(1,2)} \rightarrow \pi_{dpyb}$*	2.454
**S_2**	HOMO-1→LUMO (95%)	MXLCT $d_{Pt(1,2)} + d_{Ag} + \pi_{C\equiv CAr(1,2)} + \pi_{dpyb} \rightarrow \pi_{dpyb}$*	2.704
**S_3**	HOMO-2→LUMO (91%)	MXLCT $d_{Pt(1,2)} + d_{Ag} + \pi_{C\equiv CAr(1,2)} + \pi_{dpyb} \rightarrow \pi_{dpyb}$*	2.822
**T_1**	HOMO→LUMO (93%)	MXLCT $d_{Pt(1,2)} + d_{Ag} + \pi_{C\equiv CAr(1,2)} \rightarrow \pi_{dpyb}$*	2.406
**T_2**	HOMO-1→LUMO (65%) HOMO-2→LUMO (18%)	MXLCT $d_{Pt(1,2)} + d_{Ag} + \pi_{C\equiv CAr(1,2)} + \pi_{dpyb} \rightarrow \pi_{dpyb}$*	2.465
**T_3**	HOMO-3→LUMO (46%) HOMO-2→LUMO (14%) HOMO-1→LUMO+3 (13%)	MXLCT $d_{Pt(1,2)} + d_{Ag} + \pi_{C\equiv CAr(1,2)} + \pi_{dpyb} \rightarrow \pi_{dpyb}$*	2.617

Table S7. Characteristics of the three lowest singlet and triplet excitations at the T_1 geometry of **4b**.

State	Orbital pairing	Character	Energy, eV
**S_1**	HOMO→LUMO (95%)	MXLCT<br>$d_{Pt(1,2)} + d_{Ag} + \pi_{C\equiv CAr(1,2)} \rightarrow \pi_{dpyb}*$	2.408
**S_2**	HOMO-1→LUMO (81%)<br>HOMO-2→LUMO (14%)	MXLCT<br>$d_{Pt(1,2)} + d_{Ag} + \pi_{C\equiv CAr(1,2)} + \pi_{dpyb} \rightarrow \pi_{dpyb}*$	2.665
**S_3**	HOMO-2→LUMO (83%)<br>HOMO-1→LUMO (11%)	MXLCT<br>$d_{Pt(1,2)} + d_{Ag} + \pi_{C\equiv CAr(1,2)} + \pi_{dpyb} \rightarrow \pi_{dpyb}*$	2.767
**T_1**	HOMO→LUMO (87%)	MXLCT<br>$d_{Pt(1,2)} + d_{Ag} + \pi_{C\equiv CAr(1,2)} \rightarrow \pi_{dpyb}*$	2.356
**T_2**	HOMO-2→LUMO (57%)<br>HOMO-1→LUMO (22%)	MXLCT<br>$d_{Pt(1,2)} + d_{Ag} + \pi_{C\equiv CAr(1,2)} + \pi_{dpyb} \rightarrow \pi_{dpyb}*$	2.457
**T_3**	HOMO→LUMO+4 (71%)<br>HOMO-1→LUMO+4 (16%)	MXLCT + LC<br>$d_{Pt(1,2)} + d_{Ag} + \pi_{C\equiv CAr(1,2)} \rightarrow \pi_{C\equiv CAr(2)}*$	2.510

Table S8. Characteristics of the three lowest singlet and triplet excitations at the T_1 geometry of **4c**.

State	Orbital pairing	Character	Energy, eV
**S_1**	HOMO→LUMO (96%)	MXLCT<br>$d_{Pt(1)} + d_{Ag} + \pi_{C\equiv CAr(1)} \rightarrow \pi_{dpyb(1,2)}^*$	2.374
**S_2**	HOMO-1→LUMO (90%)	MXLCT<br>$d_{Pt(2)} + d_{Ag} + \pi_{C\equiv CAr(2)} + \pi_{dpyb(2)} \rightarrow \pi_{dpyb(1,2)}^*$	2.617
**S_3**	HOMO→LUMO+1 (95%)	MXLCT<br>$d_{Pt(1)} + d_{Ag} + \pi_{C\equiv CAr(1)} \rightarrow \pi_{dpyb}^*$	2.779
**T_1**	HOMO→LUMO (62%)<br>HOMO-1→LUMO (13%)<br>HOMO→LUMO+5 (8%)	MXLCT + LC<br>$d_{Pt(1,2)} + d_{Ag} + \pi_{C\equiv CAr(1,2)} + \pi_{dpyb(2)} \rightarrow \pi_{dpyb(1,2)}^* + \pi_{C\equiv CAr(1)}^*$	2.267
**T_2**	HOMO-1→LUMO (40%)<br>HOMO→LUMO+5 (37%)	MXLCT + LC<br>$d_{Pt(1,2)} + d_{Ag} + \pi_{C\equiv CAr(1,2)} + \pi_{dpyb(2)} \rightarrow \pi_{dpyb(1,2)}^* + \pi_{C\equiv CAr(1)}^*$	2.355
**T_3**	HOMO→LUMO+5 (38%)<br>HOMO→LUMO (28%)<br>HOMO-1→LUMO (19%)	MXLCT + LC<br>$d_{Pt(1,2)} + d_{Ag} + \pi_{C\equiv CAr(1,2)} + \pi_{dpyb(2)} \rightarrow \pi_{dpyb(1,2)}^* + \pi_{C\equiv CAr(1)}^*$	2.444

Table S9. Characteristics of the three lowest singlet and triplet excitations at the T_1 geometry of **8'**.

State	Orbital pairing	Character	Energy, eV
**S_1**	HOMO→LUMO (99%)	MXLCT $d_{Pt(1,2)} + d_{Ag} + \pi_{C≡CAr(1,2)} + \pi_{dpyb(1,2)} \rightarrow \pi_{dpyb}^*$	2.443
**S_2**	HOMO→LUMO+1 (98%)	MXLCT $d_{Pt(1,2)} + d_{Ag} + \pi_{C≡CAr(1,2)} + \pi_{dpyb(1,2)} \rightarrow \pi_{dpyb}^*$	2.737
**S_3**	HOMO-1→LUMO (96%)	MXLCT $d_{Pt(1,2)} + d_{Ag} + \pi_{C≡CAr(1,2)} + \pi_{dpyb(1,2)} \rightarrow \pi_{dpyb}^*$	2.820
**T_1**	HOMO→LUMO (91%)	MXLCT $d_{Pt(1,2)} + d_{Ag} + \pi_{C≡CAr(1,2)} + \pi_{dpyb(1,2)} \rightarrow \pi_{dpyb}^*$	2.223
**T_2**	HOMO→LUMO+1 (85%)	MXLCT $d_{Pt(1,2)} + d_{Ag} + \pi_{C≡CAr(1,2)} + \pi_{dpyb(1,2)} \rightarrow \pi_{dpyb}^*$	2.433
**T_3**	HOMO-1→LUMO (61%) HOMO→LUMO+3 (22%)	MXLCT $d_{Pt(1,2)} + d_{Ag} + \pi_{C≡CAr(1,2)} + \pi_{dpyb(1,2)} \rightarrow \pi_{dpyb}^*$	2.502

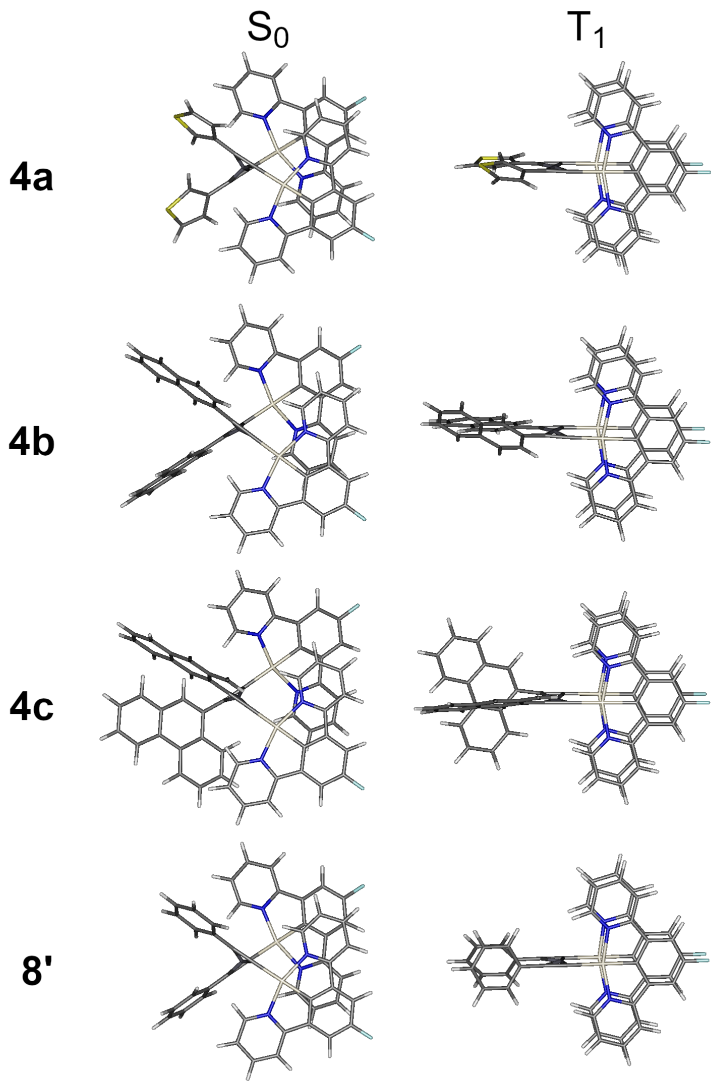

Figure S15. Ground state (S_0) and triplet excited state (T_1) geometries of complexes **4a-4c** and model complex **8'**.

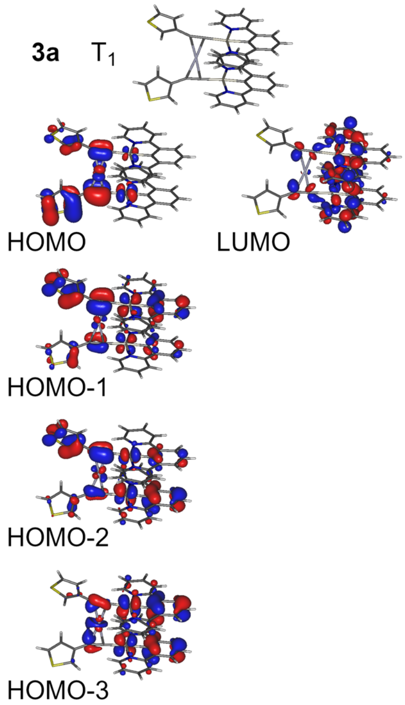

Figure S16. Molecular geometry at the T_1 state of complex **3a** and the molecular orbitals relevant to the three lowest singlet and triplet excited states.

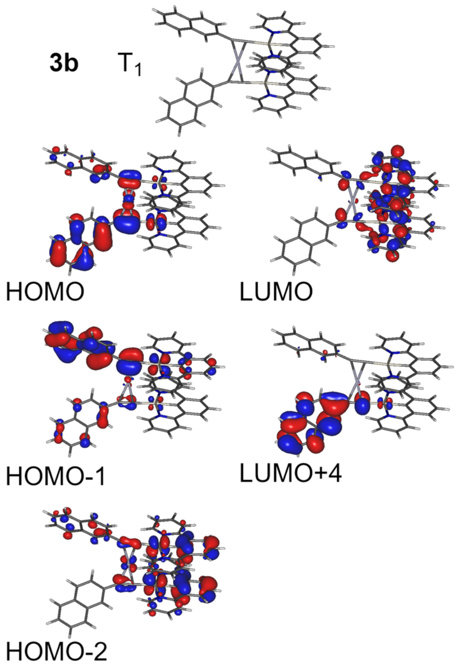

Figure S17. Molecular geometry at the T_1 state of complex **3b** and the molecular orbitals relevant to the three lowest singlet and triplet excited states.

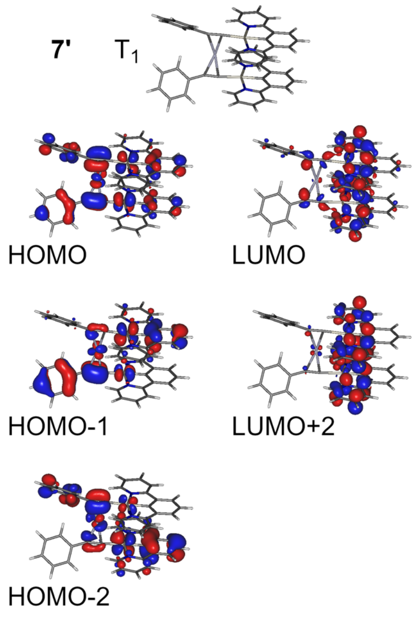

Figure S18. Molecular geometry at the T_1 state of complex **7'** and the molecular orbitals relevant to the three lowest singlet and triplet excited states.

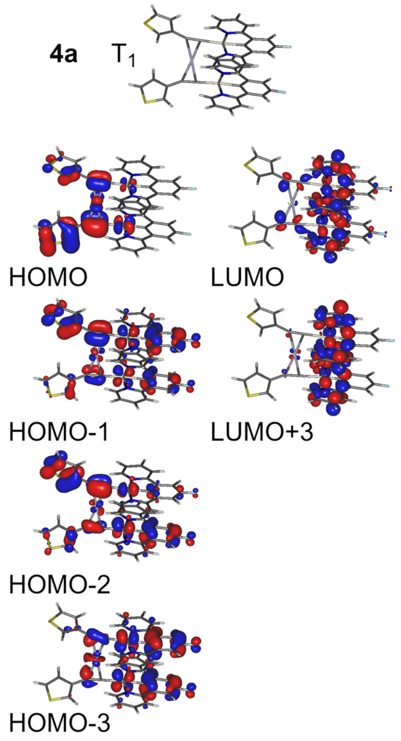

Figure S19. Molecular geometry at the T_1 state of complex **4a** and the molecular orbitals relevant to the three lowest singlet and triplet excited states.

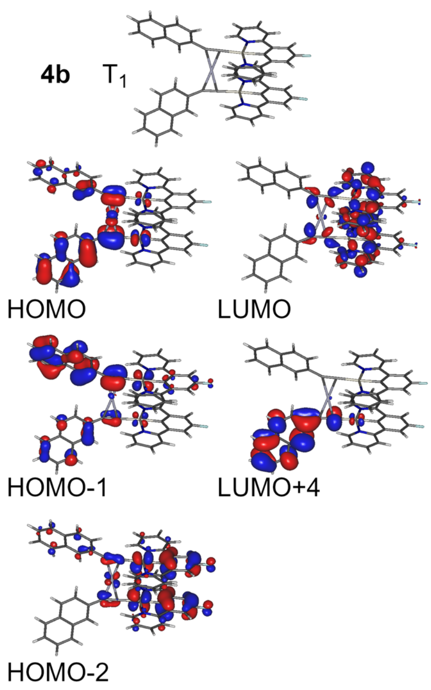

Figure S20. Molecular geometry at the T_1 state of complex **4b** and the molecular orbitals relevant to the three lowest singlet and triplet excited states.

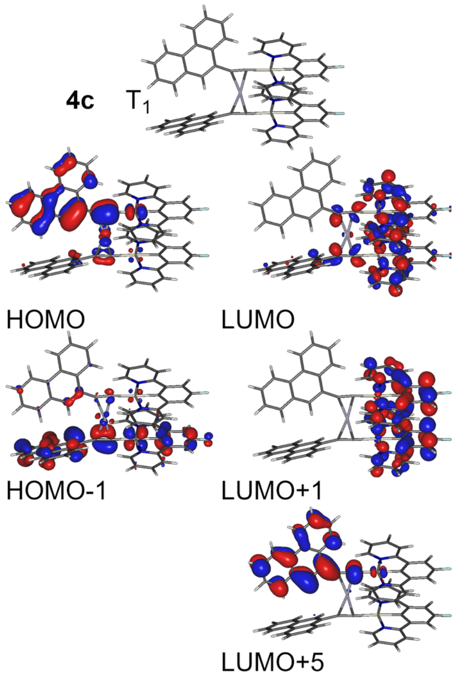

Figure S21. Molecular geometry at the T_1 state of complex **4c** and the molecular orbitals relevant to the three lowest singlet and triplet excited states.

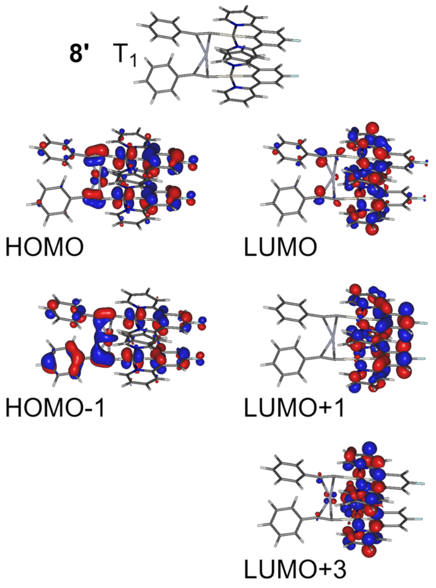

Figure S22. Molecular geometry at the T_1 state of complex **8'** and the molecular orbitals relevant to the three lowest singlet and triplet excited states.

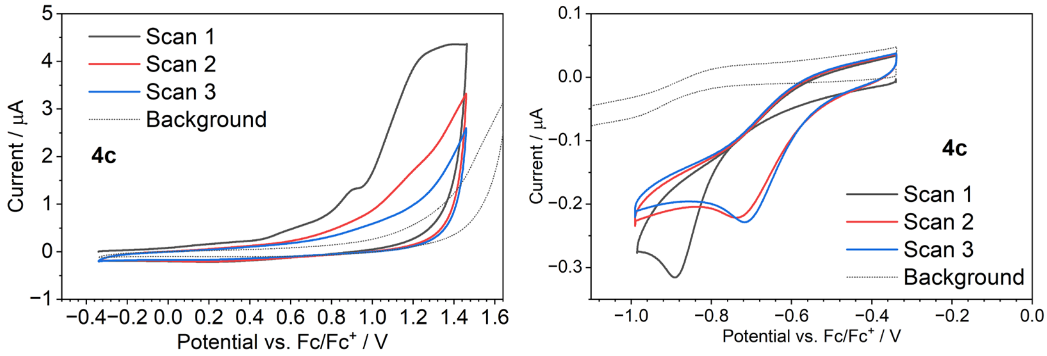

Figure S23. Cyclic voltammograms of the electrochemical response of **4c** at 0.5 mM in 0.1 M tetrabutylammonium tetrafluoroborate in CH_2Cl_2.

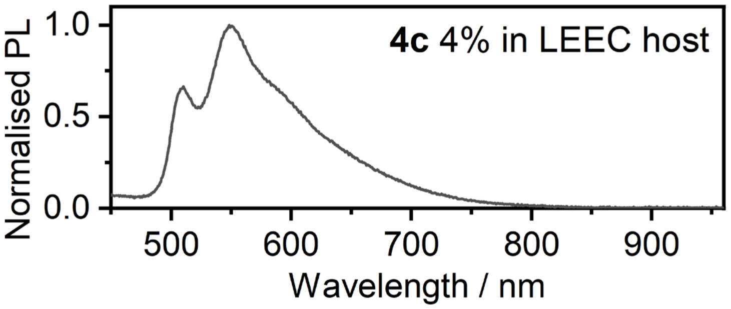

Figure S24. PL spectrum of **4c** doped into LEEC host PVK:OXD7:THABF$_4$ (56:38:6) recorded at nitrogen atmosphere.

4 References

1 J. R. Lakowicz, *Principles of Fluorescence Spectroscopy*, Springer US, 2007.

2 O. Elbjeirami, M. A. Rawashdeh-Omary and M. A. Omary, *Res. Chem. Intermed.*, 2011, **37**, 691–703.
